# *Ipomoea batatas* L. Lam. ameliorates acute and chronic inflammations by suppressing inflammatory mediators, a comprehensive exploration using in vitro and in vivo models

**DOI:** 10.1186/s12906-018-2279-5

**Published:** 2018-07-13

**Authors:** Muhammad Majid, Bakht Nasir, Syeda Saniya Zahra, Muhammad Rashid Khan, Bushra Mirza, Ihsan-ul Haq

**Affiliations:** 1 Department of Pharmacy, Faculty of Biological Sciences, Quaid-i-Azam University, Islamabad, 45320 Pakistan; 2 Department of Biochemistry, Faculty of Biological Sciences, Quaid-i-Azam University, Islamabad, 45320 Pakistan

**Keywords:** Inflammation, Arthritis, Sweet potato, *Ipomoea*, CFA, Antioxidant, Interleukin

## Abstract

**Background:**

*Ipomoea batatas* L. Lam. is a functional food and belongs to family Convolvulaceae. It is used as an antiinflammatory, aphrodisiac, antiasthmatic, anticonvalescent, antitumor, antanemic and antidiabetic agent by local communities. This study has been planned to evaluate its antiinflammatory and antiarthritic potentials.

**Methods:**

Dry powder of *I. batatas* tuber and roots were extracted with ethyl acetate (IPT-EA, IPR-EA) and methanol (IPT-M, IPR-M), respectively. These extracts were tested for total phenolic and flavonoid contents (TPC and TFC), HPLC finger printing, multidimensional in vitro and in vivo antioxidant potential and albumin denaturation inhibition. Carrageenan-induced paw edema, croton oil-induced ear and anal edema inhibition and Complete Freund’s Adjuvant (CFA)-induced antiarthritic assays were executed at a dose of 300 mg/kg body weight on Sprague-Dawley rats. Serum levels of interleukins IL-1β and IL-6 and nitric oxide (NO) were assessed to measure the inhibition of inflammation.

**Results:**

Maximal TPC (319.81 ± 14.20 μg GAE/mg dry extract) and TFC (208.77 ± 9.09 μg QE/mg DE) were estimated in IPR-EA extract. IPT-EA and IPR-EA yielded the maximum amounts of rutin (7.3 ± 1.12 and 4.5 ± 0.55), caffeic acid (1.60 ± 0.25 and 2.17 ± 0.26) and myricetin (2.7 ± 0.14 and 1.01 ± 0.08 μg/mg DE), respectively in HPLC-DAD analysis. All extracts showed dose dependent response in in vitro antioxidant assays. Best inhibition (76.92 ± 3.07%) of albumin denaturation was shown by IPT-EA in comparison to ibuprofen (79.48 ± 4.71%). IPR-EA exhibited highest edema inhibition in models of carrageenan-induced paw edema (79.11 ± 5.47%) and croton oil-induced ear and anal edema (72.01 ± 7.80% and 70.80 ± 4.94%, respectively). Significant inhibition of CFA-induced arthritic edema and arthritic score were observed by IPR-EA as compared to ibuprofen. Suppression of pro-inflammatory cytokines (IL-1β, IL-6) and NO levels was shown by IPR-EA and IPT-EA, respectively.

**Conclusion:**

These results depict that richness of polyphenols and phytoconstituents in *I. batatas* ameliorates oxidative stress and inflammation of acute and chronic nature. Dose dependent antioxidant potential and inhibition of inflammatory edema, pro-inflammatory cytokines and hematological, biochemical and histological changes prove *I. batatas* therapeutic potential as an antiinflammatory and antiarthritic agent.

**Graphical abstract:**

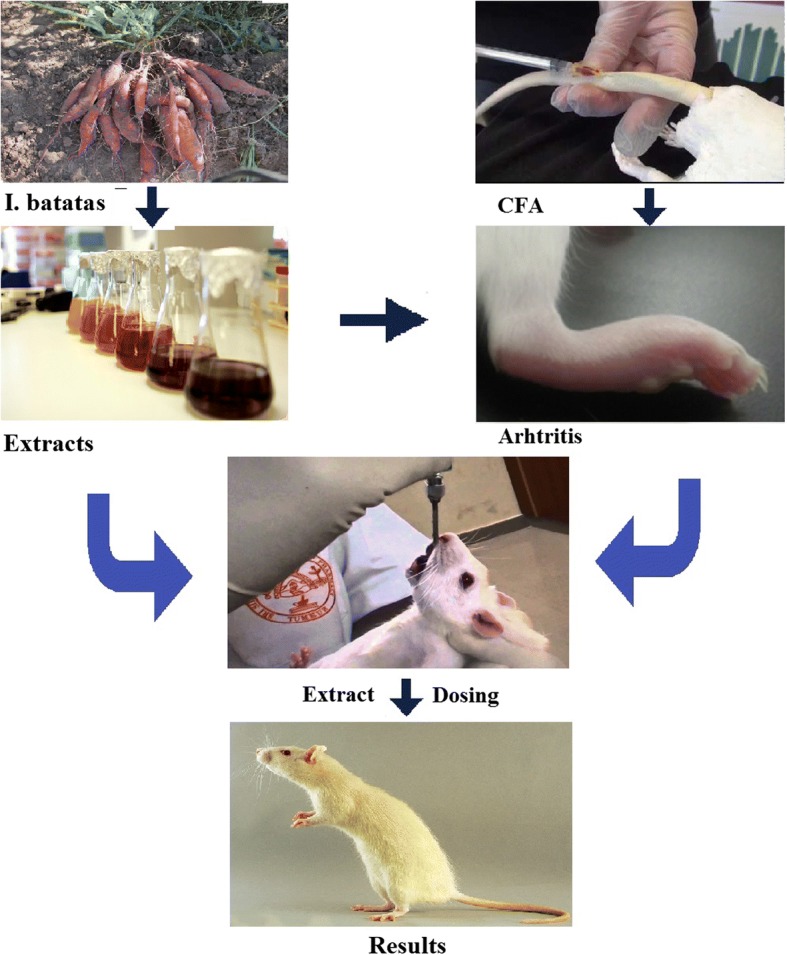

**Electronic supplementary material:**

The online version of this article (10.1186/s12906-018-2279-5) contains supplementary material, which is available to authorized users.

## Background

Inflammation is considered to be an important causative agent of morbidity and mortality associated with a range of diseases. Inflammation involves several metabolic and cellular events coordinated by mediators like cytokines, interleukins (IL), prostaglandins (PGs) and thromboxanes. A number of inflammatory disorders such as rheumatoid arthritis (RA), several carcinomas, atherosclerosis and asthma have high prevalence worldwide [1]. Rheumatoid arthritis, a chronic inflammatory problem, is an auto-immune disorder responsible for series of erratic articular cartilage and subcondral bone damages [2]. It involves several cellular and metabolic events involving profuse amount of mediators like interleukins, cytokines, thromboxanes and prostaglandins [3]. Although, it is a protective effort of immune system of the organism to eradicate the invading pathogens and initiate the process of healing in stressed tissues [4]; however; unrestrained inflammation may lead to the beginning of several diseases like rheumatoid arthritis, atherosclerosis and vasomotor rhinorrhea [1]. Though several molecules like tumor necrosis factor (TNF), IL and PGs are the causative agents of RA pathology [5] but reactive oxygen species (ROS) are much notorious for stimulating inflammatory pathways via their harmful attitude towards macromolecules of the cells [6].

Reactive nitrogen species (RNS) and ROS are continuously produced as byproducts during the routine metabolic processes [7, 8]. According to the circumstances and situations, free radicals act as dual-edged sword either friend or foe. When friendly, they carry important jobs in neurotransmission, defense against microbes and cytotoxicity [9] but in excess, these lead to oxidative stress, which is the root cause of many chronic inflammatory disorders such as rheumatism, diabetes, cancer, aging and circulatory disorders [10]. ROS invades and damages endothelial cells with increase in the permeability of the microvasculature facilitating the migration of neutrophils to the foci of inflammation [11]. Extensive experimental investigations concerning assays of nitro-tyrosine residues in synovial fluids of RA patients or in vitro exposure of chondrocytes to peroxynitrite concluded that conversion of superoxide anions to NO leads to cartilage abrasion. Several endogenous detoxifying enzymes and metabolites provide first line of defense inside the living system to eliminate the deleterious effects of free radicals [12]. Catalase (CAT), peroxidase (POD), and superoxide dismutase (SOD) are the notable and significant detoxifying enzymes.

Diseases of cartilage abrasions such as rheumatoid and osteoarthritis (OA) involve unstable phenotype of differentiated chondrocytes, localize inflammation, progressive cartilage abrasion, aching and joints tenderness [13–15]. Assessment of cytokines, chemokines and NO [16–18] levels are helpful in finding degrees of arthritic progression. Most commonly practiced therapy for these inflammatory disorders are non-steroidal antiinflammatory drugs (NSAIDs), as they are good at reducing pain, redness, edema and fever. But these drugs are accompanied with numeral adverse effects of supreme severity in case of multi-dose treatment [19]. Hence, the use of natural substances including food plants with medicinal virtues is getting more attention for arthritis management.

Efficient food products are intended to introduce human dietary constituents that aid specific physiological functions in addition to being nutritive. Sweet potato (*Ipomoea batatas* L. Lam.), the 7th most important food crop in the world (FAO 1997) [20] and a member of family Convolvulaceae, is an imperative root vegetable with large size, high starch content and sweet taste [21]. It is used in folk medicine as aphrodisiac, antiinflammatory, astringent, demulcent, energizer, laxative, bactericidal and antifungal agent. It’s also helpful in treating mouth and throat tumors, asthma, bug bites, burns, catarrh, diarrhea, fever, nausea, splenosis, stomach distress [22], anemia, hypertension, diabetes and prostatitis [23]. Several phytochemical studies of *I. batatas* have detected numerous potential compounds with profound medicinal worth like vitamins such as pantothenic acid (vitamin B5), pyridoxine (vitamin B6), and thiamin (vitamin B1), as well as niacin, riboflavin [21], β-carotene, iron, calcium, zinc, protein [24], polyphenols like anthocyanin and phenolic acids such as caffeic, monocafeoyl quinic, dicaffeoylquinic, and tricaffeoylquinic acids [25, 26]. The tubers are packed with many essential vitamins.

Functional food likeness, non-toxic attitude and antiinflammatory potential of *I. batatas* make it a strong candidate to be assessed as a natural remedy to prevent and control inflammatory diseases. Literature review demonstrates its antiinflammatory potential but the studies performed were not comprehensive and no antiarthritic study on any part of the plant has been conducted. Therefore, multi-mode validation of the potency in terms of antiinflammatory prospective was needed. Scientists have been using tubers and veins of *I. batatas* for the investigation of several biological and phytochemical inquiries. We, for the first time, have used *I. batatas* roots along with tuber to inquire its phytochemical and biological aptitude. A detailed in vitro and in vivo study was conducted to prove antirheumatoid arthritis abilities of roots and tubers of *I. batatas*.

## Methods

### Chemicals and reagents

Complete Freund’s Adjuvant (CFA), carrageenan, ibuprofen, tween 80, ascorbic acid, 2,2- diphenyl-1-picrylhydrazyl (DPPH), thiobarbituric acid, ferric chloride, aluminum chloride, 2,2-azino-bis-(3-ethylbenzothiazoline-6-sulphonic acid) diammonium salt, potassium persulphate, trichloroacetic acid, phenazine methosulphate, and Folin–Ciocalteu reagent were purchased from Sigma (Chemicals Co. St. Louis, USA). Disodium hydrogen phosphate, sodium dihydrogen phosphate, hydrogen peroxide, sodium hydroxide, sodium carbonate, sodium nitrite, potassium ferricyanide, sulphuric acid, deoxyribose, ferrous chloride were purchased from Merck KGaA Darmstadt Germany. All other chemicals were obtained from Sigma (Chemicals Co. St. Louis, USA). All the chemicals were of analytical grade.

### Preparation of extract

Plant was recognized by its local name and collected from the District Sargodha in January 2016. The plant sample was identified and authenticated by Prof. Dr. Rizwana Aleem Qureshi, Department of Plant Sciences, Faculty of Biological Sciences, Quaid-i-Azam University, Islamabad, Pakistan. It was then deposited (Accession No. PHM-501) at the Herbarium of Pakistan, Quaid-i-Azam University. Tubers and roots of *I. batatas* were washed with fresh water and air dried with no exposure to direct sunlight. Fully shade dried parts of the plant were first powdered followed by successive extraction with n-hexane, ethyl acetate, methanol and distilled water. These extracts were dried under vacuum in a rotary evaporator at 40 °C and evaluated for several bench top assays. On the bases of high phenolic and flavonoid contents and significant antioxidant activity ethyl acetate (IPT-EA) and methanol extracts (IPT-M) from tubers while ethyl acetate (IPR-EA) and methanol extracts (IPR-M) from roots were selected for further in vitro and in vivo experiments.

### Phytochemical and antioxidant profiling

#### Reckoning the total phenolic content (TPC)

The total phenolic contents were estimated according to slightly modified procedure as described previously using Folin–Ciocalteu reagent [27]. An aliquot of 20 μl from 4 mg/ml DMSO stock solution of each test sample was transferred in respective well of 96-well plate followed by addition of 90 μl of Folin–Ciocalteu reagent. The plate was incubated for 5 min after which 90 μl of sodium carbonate was added to the reaction mixture. Gallic acid was used as standard and absorbance of each reaction mixture was taken at 630 nm using microplate reader (Biotech USA, microplate reader Elx 800) in triplicate. Gallic acid (6.25–50 μg/ml) was used as a positive control and the results are expressed as μg gallic acid equivalent per mg dry extract (μg GAE/mg DE).

#### Estimation of total flavonoid content (TFC)

For total flavonoid content determination, aluminum chloride colorimetric method was employed with slight modifications according to system suitability [28]. The crude extracts (20 μl of 4.0 mg/ml in DMSO) were transferred to each well of 96-well plate. Subsequently, 10 μl of each 10% aluminum chloride and 1.0 M potassium acetate was added followed by the addition of 160 μl of distilled water. The resulting mixture was kept at room temperature for 30 min. Then absorbance of the plate was measured at 415 nm using microplate reader. The calibration curve was drawn by using quercetin as standard at final concentrations of 0, 2.5, 5, 10, 20, 40 μg/ml. The resultant flavonoid contents were calculated as quercetin equivalent per mg dry extract (μg QE/mg DE).

#### High performance liquid chromatography (HPLC) exploration

For the detection and quantification of polyphenols, HPLC-DAD analysis was conducted following the methodology of Sahreen et al. [29] with little modifications. HPLC analysis of IPT-EA, IPT-M, IPR-EA and IPR-M was carried out by using HPLC-DAD (Agilent Germany) equipment using Zorbax RX-C8 (4.6 × 250 mm, 5 μm particle size, Agilent, USA). Mobile phase consisted of eluent A, (acetonitrile:methanol:water:acetic acid/ 5:10:85:1) and eluent B (acetonitrile:methanol:acetic acid/ 40:60:1). Following gradient (A:B) was utilized: 0–20 min (0 to 50% B), 20–25 min (50 to 100% B), and isocratic 100% B (25–40 min) at flow rate of 1 ml/min. The injection volume of the sample was 20 μl. Before the injection, samples were filtered through 0.45 μm membrane filter. Among the standards, rutin was analyzed at 257 nm, gallic acid and catechin at 279 nm, caffeic acid and apigenin at 325 nm while quercetin, myricetin and kaempferol were analyzed at 368 nm. Each time the column was reconditioned for 10 min before the next analysis. All samples were assayed in triplicates. Quantification was carried out by the integration of the peak using the external standard method. All chromatographic operations were carried out at an ambient temperature.

### Determination of in vitro antioxidant activity

The different concentrations of sample ranging from 0 to 500 μg/ml were used in all in vitro antioxidant tests. The stock solution of the sample was prepared by mixing 4 mg in 1 ml of DMSO and dilutions were made for each in vitro antioxidant assay. Standard compounds were used for the comparisons of antioxidant potential of the sample.

The antioxidant potential of IPT-EA, IPR-EA, IPT-M and IPR-M extracts against DPPH, nitric oxide, hydroxyl radical and iron chelation capacity was determined following the previous protocols [29]. The scavenging activity was calculated using the following equation:

$$ \%\mathrm{scavenging}\ \mathrm{activity}=1-\left(\mathrm{OD}\ \mathrm{of}\ \mathrm{sample}\right)/\left(\mathrm{OD}\ \mathrm{of}\ \mathrm{control}\right)\times 100 $$

Reducing power (TRP) and total antioxidant capacity (TAC) was determined by themethodology illustrated by Phull et al. [30]. The results were expressed as μg QE/mg DE.

### Animal ethical statement

The experiment was conducted by strictly following the guidelines approved by the ethical committee of Quaid-i-Azam University, Islamabad, Pakistan (Letter No. QAU-PHM-017/2016 for the animal care and Letter No. QAU-PHM-023/2016 dated 24/10/2016 for experiments). Blood sampling from healthy volunteers was also approved by Institutional Review board of Quaid-i-Azam University (Letter No. IRB-QAU-116 dated 4/11/2016). The study followed ethical guidelines of minimal distress, discomfort and pain to the subject animals ensuring that experiments provided new knowledge or lead for human/animals wellbeing. Appropriate sedation, analgesia and anesthesia techniques were used where necessary as well as informed consent was obtained from human subject. Euthanasia was done by cervical dislocation under chloroform anesthesia.

### Experimental animals

Male Sprague-Dawley rats having weight of ~ 150–200 g were used for this study and were kept in cages (made up of aluminum) under controlled conditions (12 h light/dark cycle, 25 ± 1 °C temperature). All the animals were supplied with standard feed along with water ad libitum prior to use in experiments. Animals were divided into seven groups and each group comprised of 6 male Sprague-Dawley rats for each treatment. Specified doses of *I. batatas*, positive and negative controls were orally administered to each group as follows;

- Group-I: IPT- EA (300 mg/Kg)
- Group-II: IPT-M (300 mg/Kg)
- Group-III: IPR- EA (300 mg/Kg)
- Group-IV: IPR-M (300 mg/Kg)
- Group-V: Positive (ibuprofen 10 mg/Kg)
- Group-VI: Disease control
- Group-VII: Normal

### Toxicity assessment

#### Isolation and monolayer culture of rabbit articular chondrocytes

Three weeks old local strain of rabbits of either sex at Primate Facility of Faculty of Biological Sciences, Quaid-i-Azam University, Islamabad were sacrificed and articular cartilage pieces were collected. Thereafter, chondrocytes were isolated from cartilage slices using collagenase type II (381 units/ml) for 12 h at 37 °C in CO_2_ incubator. The cells were cultured in DMEM medium supplemented with 10% bovine calf serum, streptomycin (50 μg/ml), and penicillin (50 unit/ml). The chondrocytes were seeded at density of 1× 10^4^ cells per well in 96-well plate for toxicity and viability assay. Culture media was replaced every 2 days and cells at 75% confluence were used for further study [30].

#### Isolation of lymphocytes from blood

Lymphocytes were isolated from human blood using previously described protocol with some modifications [31, 32]. A volume of 3 ml of blood was obtained from a healthy donor by venipuncture and diluted (1,1) with PBS. It was layered over 2 ml Histopaque-1077 and centrifuged at 800 X g for 20 min. The buffy coat was aspirated into 5 ml of PBS and centrifuged at 350 rpm for 4 min to pellet the lymphocytes. The pellet was suspended in 1 ml of RPMI-1640 and cell density was adjusted to get 1 × 10^5^ cells/ml.

#### In vitro toxicity assessment on rabbit articular chondrocytes

The methylthiazole tetrazolium (MTT) assay and microscopic observation were performed to evaluate the cytotoxic effects of *I. batatas* on rabbit articular chondrocytes following previously described protocol [13]. Initially, 1 × 10^4^ chondrocytes were seeded in each well (three wells for each dose) and incubated in a CO_2_ incubator overnight for attachment. Thereafter, cells were exposed to various concentrations (0 to 1000 μg/ml) of test sample for 24 h. Then, 10 μl of reagent 1 (methyl tetrazolium, 1%) was added in each well and kept in a CO_2_ incubator for 4 h until purple formazan crystals developed. Followed by the addition of 100 μl of reagent 2 (solubilization buffer, 10% SDS with 0.01 N HCl and DMSO) the plate was incubated in a CO_2_ incubator for 12 h after which absorbance was measured at 590 nm using a Versa-Max microplate reader (Molecular Devices Co., Sunnyvale, CA, USA). The experiment was performed in triplicate.

#### In vitro toxicity assessment on blood lymphocytes

To assess genotoxicity*,* comet assay was performed with little modifications in the previously described protocol [33]. Briefly, 20 μl of samples (100 μg/ml) or ethyl methane sulfonate (20 μg/ml) or 1% DMSO in PBS and 180 μl of lymphocyte suspension were incubated in 96-well plate at 37 °C for 3 h in humidified 5% carbon dioxide incubator (Panasonic, Japan MCO-18 AC-PE). After 3 h, the cells were centrifuged to pellet, washed with PBS and 0.5% LMA at 37 °C was added in cells. Then 75 μl of this suspension containing approximately 1000 cells was plated on frozen slides pre-coated with 1% NMA and a cover slip was gently placed over it. The slide was placed on icepacks for about 8–10 min. Cover slip was detached and again LMA was added and placed on ice packs for solidification. After three coatings with low melting point agarose, electrophoresis was performed and 1% ethidium bromide was used to stain. The slides were visualized under fluorescent microscope and CASP 1.2.3.b image analysis software was used to evaluate the extent of DNA damage. In each sample, 50–100 cells were analyzed for comet length, head length, tail length, tail moment, DNA content in head and tail of lymphocytes.

#### In vivo acute toxicity assessment in rats

For the acute toxicity assay, rats were randomly divided into control and test groups (*n* = 6). The test groups were treated with increasing doses of 300, 500, 1000, 2000, and 4000 mg/kg of test samples. The toxic symptoms, mortality rates and behavioral pattern such as lethargy, salivation, lacrimation, nasal secretions, balance, mood and aggression, piloerection, frequency of urination and defecation, sleep, symptomatic observation for any injury and pain were observed on daily basis for two weeks after intragastric administration of the sample. Control group was administered with saline (10 ml/kg of animals) and toxicity assessment was executed by following the Organization for Economic Cooperation and Development (OECD) guidelines 425.

### Antiinflammatory and antiarthritic evaluation

#### In vitro antiinflammatory activity

The antiinflammatory efficacy was gauged by inhibition of albumin denaturation procedure with minor amendments in methodology of Leelaprakash et al. [34]. The test extracts were incubated with 1% aqueous solution of bovine albumin fraction at 37 °C for 20 min having pH 7.4. After incubation, samples were heated to to 51 °C for 20 min, cooled and turbidity was measured at 660 nm. Experiment was repeated thrice and the % inhibition of protein denaturation was calculated as follows:

$$ \mathrm{Percentage}\ \mathrm{inhibition}=\left(\mathrm{Abs}\ \mathrm{Control}-\mathrm{Abs}\ \mathrm{Sample}\right)\ \mathrm{X}\ 100/\mathrm{Abs}\ \mathrm{control} $$

#### Test sample and standard drug for in vivo analysis

Solutions of samples were prepared by dissolving in 10% DMSO at dose of 300 mg/kg body weight (BW) per rat and standard drug ibuprofen was prepared at dose of 10 mg/kg BW. Test sample and positive control were orally administered. Four extracts of *I. batatas* (IPT-EA, IPT-M, IPR-EA and IPR-M) were used in the present study.

#### Carrageenan-induced inflammation impediment in rats

The antiinflammatory competence of *I. batatas* was appraised with the help of carrageenan-induced rat paw edema model [10]. Among seven groups of experimental animals (Sprague-Dawley rats) as described above, Group I-IV were test groups, i.e., IPT-EA, IPT-M, IPR-EA and IPR-M extracts at the dose of 300 mg/kg BW, Group V was positive control ibuprofen at the dose of 10 mg/kg BW, Group VI was carrageenan control and Group VII was saline control, which received 0.1 ml normal saline. The drugs were administered an hour before injection of 150 μl of carrageenan suspension (0.9% *w*/*v* in saline) in left hind paw of each rat for the duration of one day. The paw volume was measured at different time periods (1, 3, 6, 12, 24 h) by using Plethysmometer (Ugo Basile 7140, Italy). Results were calculated as.

$$ \mathrm{Edema}\ \mathrm{volume}=\mathrm{PVt}-\mathrm{PVc} $$

where PVt is paw volume (ml) at specific time after carrageenan administration while PVc is paw volume (ml) before carrageenan administration.

$$ \mathrm{Percent}\ \mathrm{inhibition}=\mathrm{EVc}-\mathrm{EVt}/\mathrm{EVc}\times 100 $$

where EVc is edema volume of control group. EVt is edema volume of treated group.

#### Croton oil-induced ear edema constraint

Method described by Reanmongkol et al. was used with minor modifications [35]. Cutaneous inflammation was induced by applying 100 μl of acetone solution containing the irritant (5% croton oil) to the inner surface of the right ear of the mice. Acetone was applied to the left ear as vehicle. IPT-EA, IPT-M, IPR-EA and IPR-M (5.0 mg/ear each) were applied topically to the right ear 1 h before application of croton oil. Ibuprofen (1 mg/ear) served as a reference antiinflammatory drug. A plug (7-mm diameter) was removed from both the treated and untreated ears after sacrificing the rats four hours later. The % inhibition of edema was calculated as the difference of weight in two plugs.

#### Croton oil-induced anus edema inhibition

Croton oil-induced edematous response of anus was evaluated by modifying the methodology of Reanmongkol et al. [35]. A cotton swab soaked with the inducer (0.2 ml of 6% croton oil in diethyl ether) was introduced into the anus of rats for 10 s. One hour later, vehicle control, IPT-EA, IPT-M, IPR-EA and IPR-M (300 mg/kg each), and ibuprofen (10 mg/kg) were orally given OD for 3 days to respective groups. On the fourth day, size of each anaesthetized rat’s anus (mm) was scaled with the help of a vernier caliper.

#### Complete Freund’s adjuvant-induced arthritis in rats

Arthritic rat model was prepared by using Complete Freund’s adjuvant (heated-killed Mycobacterium tuberculosis in 1 ml of liquid paraffin at 10 mg/kg). Briefly, 200 μl of CFA emulsion was injected subcutaneously at the base of the tail of rat under anesthesia [36]. Study plan was sub-divided in following two schemes.

#### Treatment mode

In this study mode, arthritis was induced till 11–13 days and IPT-EA, IPT-M, IPR-EA, IPR-M (300 mg/kg each) and ibuprofen (10 mg/kg) were given through oral route on alternate days to respective groups. Reversal of maximum arthritic symptoms was assessed.

#### Preventive mode

In this study mode, single dose CFA was injected and IPT-EA, IPT-M, IPR-EA, IPR-M (300 mg/kg each) and ibuprofen (10 mg/kg) were given concurrently through oral route on alternate days and evaluation of prevention from arthritis was done in test groups with comparison to the controls.

#### Adjuvant-induced arthritic scoring

Experimental animals were investigated on a daily basis for the signs of arthritic severity through well-established scoring system [36]. Severity of the inflammation in paws was graded on the basis of swelling, induration and erythema using 5-point scale scoring system. In this scale, no sign of diseases (non-toxic), signs involving the wrist/ankle, signs involving the ankle plus tarsal of the hind paw and/or wrist plus carpals of the fore paw, signs extending to the metatarsals or metacarpals and severe disease involving the entire hind or fore paw were assigned as 0 to 4 scores [37]. Paw volume was measured on alternate days using Plethysmometer (Ugo Basile 7140, Italy). Paw edema was monitored from the total paw volumes of 4 paws.

### Histological investigation

On the last day (25th day) of experiment, the arthritic rats were sacrificed. The dissected tissue of arthritic joint was fixed by buffered formaldehyde (10%, pH 7.4) at room temperature for 12 h. The water and infiltrated wax of fixed tissue was removed by repeated washing with ethanol. The tissue was cut into small pieces of 5 μm thickness with rotary microtome. The sections were stained with Eosin and Haematoxylin. Histological investigation was carried out under Nikon Microscope (Eclipse 80i, Japan).

### Determination of body weight and relative organ weight of rats

Rats and their major organs such as liver, kidney, spleen and thymus were weighed in grams and index of the organ or relative weight was calculated as

$$ \mathrm{ROW}=\mathrm{AOW}/{\left(\mathrm{BW}\right)}^{\ast }\ 100 $$

ROW = relative organ weight, AOW = absolute organ weight (g), BW = body weight on final day (g).

### Collection of blood sample and biochemical analysis

On the last day, experimental rats were anaesthetized by chloroform inhalation and euthanized by cervical dislocation. The blood samples were collected under anesthesia via the abdominal aorta in specific tubes (BD vacutainer) for hematological, biochemical and serological investigations. Serum was separated by centrifuging blood samples at 6000 rpm for 15 min at 4 °C that was either analyzed or stored at − 20 °C. Afterwards, the animals were dissected via a ventral longitudinal abdominal incision. Major organs, i.e., liver, spleen, thymus, and kidney were identified and dissected out for relative organ weight analysis.

### Hematological studies

The white blood cells (WBCs), red blood cells (RBC) and platelets were counted by using neubauer hemocytometer (Feinoptik, Germany). The hemoglobin (Hb) content was estimated by Sahli’s hemoglobin meter. Erythrocyte sedimentation rate was measured through the modified Westergren method [38].

### Determination of biochemical parameters

Different parameters such as urea, creatinine, alkaline phosphatase (ALP), alanine transaminase (ALT), aspartate transaminase (AST) and testosterone were determined from the sera of experimental rats using standard AMP diagnostic kits (Stattogger Strasse 31b 8045 Graz, Austria). The protein concentration was determined by the Bradford method [39].

### Determination of endogenous antioxidant enzymes of rats

Activities of serum catalase (CAT) were evaluated by monitoring the rate of H_2_O_2_ hydrolysis at 240 nm [40]. The superoxide dismutase (SOD) activity in serum was measured by quercetin autoxidation inhibition method [41]. Peroxidases (POD) were determined by following the slightly modified method as described previously [40].

### Measurement of cytokines and NO levels in serum

The levels of cytokines (IL-1β and IL-6) from serum were measured by enzyme-linked immunosorbent assay (ELISA), according to the manufacturer’s protocol (BD Biosciences, USA) while NO levels were determined by mixing serum (30 μl) with 0.3 M NaOH and 5% ZnSO4. After centrifugation for 15–20 min at 6400×g, an aliquot of 10 μl of the supernatant was mixed with 200 μl of Griess reagent in 96-well plate. The absorbance was recorded at 540 nm. Sodium nitrite curve was used to quantify nitrite amount in serum [42].

### Statistical analysis

Data obtained in this study was presented as mean ± SD. One way analysis of variance was performed to determine the variability among groups by Statistix 8.1. GraphPad Prim 5 was used to determine the correlation of IC_50_values of antioxidant assays with TPC and TFC by Pearson’s correlation coefficient. Significant differences among groups were calculated by Tukey’s multiple comparison and Kruskal-Wallis tests. Statistical significance was set at *p* < 0.05,* p *< 0.01 and *p* < 0.001.

## Results

### Phytochemical investigation

#### Total phenolic and flavonoid contents

Considering the standard regression lines for gallic acid (y = 0.0083× + 0.0182; R^2^ = 0.9766) and quercetin (y = 0.0088× + 0.0151; R^2^ = 0.9922), the equivalents of TPC and TFC were calculated (Table 1). IPR-EA showed maximum quantity of TPC (319.81 ± 14.20 μg GAE/mg DE) followed by IPT-EA (286.68 ± 4.90 μg GAE/mg DE), IPR-M (262.59 ± 5.70 μg GAE/mg DE) and IPT-M (229.45 ± 5.01 μg GAE/mg DE). Flavonoids were found to be rich in IPR-EA (208.77 ± 9.09 μg QE/mg DE) followed by IPT-EA (188.89 ± 2.40 μg QE/mg DE), IPR-M (177.81 ± 2.50 μg QE/mg DE) and IPT-M (146.27 ± 2.80 μg QE/mg DE).

**Table 1:** Total phenolic content, total flavonoid content, total antioxidant capacity and total reducing power of *I. batatas* extracts

Samples	TPC(μg GAE/mg DE)	TFC(μg QE/mg DE)	TAC(μg QE/mg DE)	TRP(μg QE/mg DE)
IPT-EA	286.68 ± 4.90^b^	188.89 ± 2.40^b^	442.48 ± 4.85^b^	332.48 ± 4.06^b^
IPT-M	229.45 ± 5.01^d^	146.27 ± 2.80^d^	361.65 ± 3.35^d^	256.09 ± 4.56^d^
IPR-EA	319.81 ± 14.20^a^	208.77 ± 9.09^a^	485.71 ± 4.26^a^	370.67 ± 5.28^a^
IPR-M	262.59 ± 5.70^c^	177.81 ± 2.50^c^	416.15 ± 5.95^c^	304.71 ± 4.42^c^

Data values shown represent mean ± SD (*n* = 3). Means with different superscript (a-d) letters in the column sare significantly (*p* < 0.05) different from one another

*TPC* total phenolic content, *TFC* total flavonoid content, *TAC* total antioxidant capacity, *TRP* total reducing power, *GAE* gallic acid equivalent, *QE* quercetin equivalent, *DE* dry extract

#### HPLC-DAD quantification

**Fig. 1:**
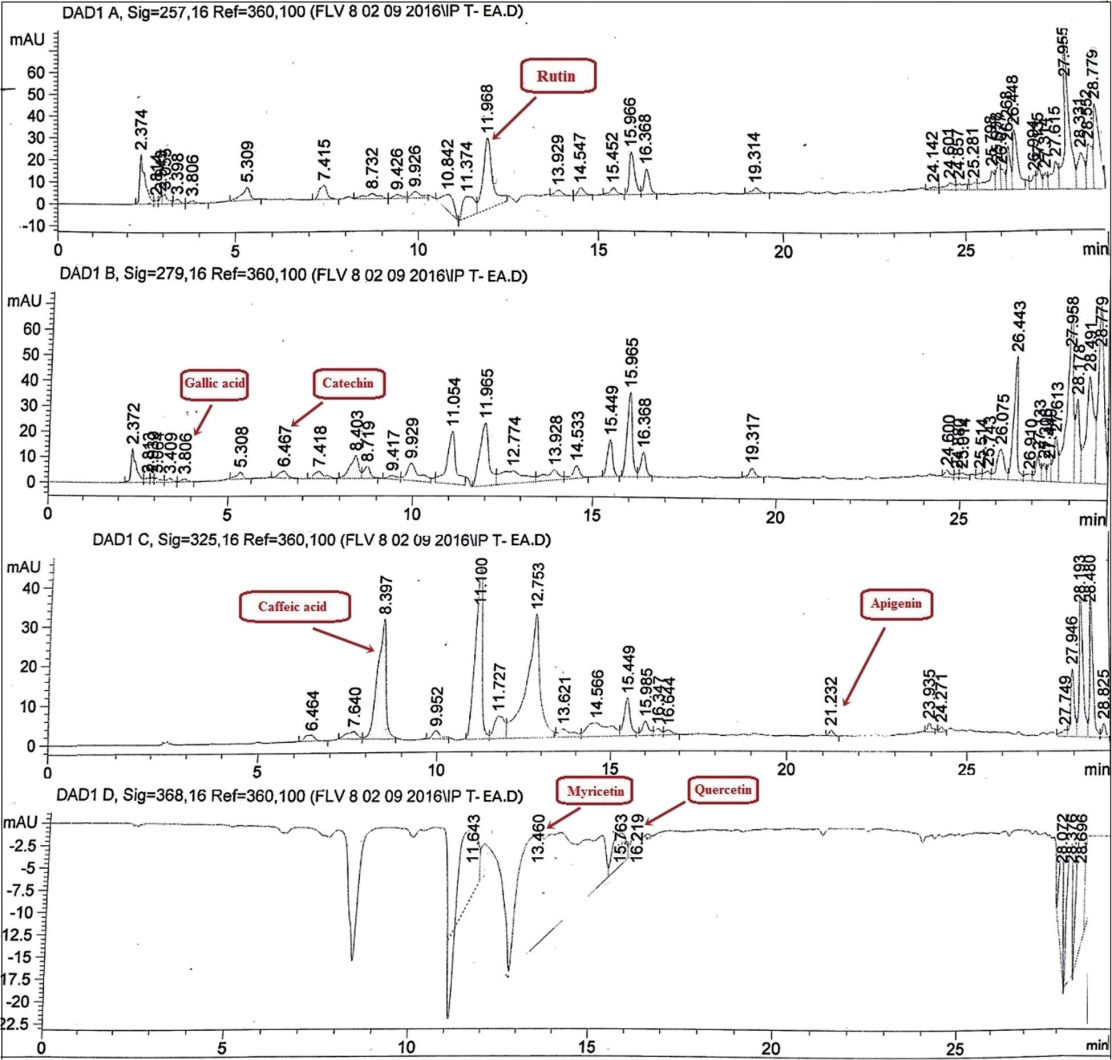
HPLC-DAD profile of *I. batatas* tuber ethyl acetate extract (IPT-EA) at different wavelengths. Signal 1: 257λ, Signal 2:279λ, Signal 3: 325λ, Signal 4; 368λ.Conditions: Mobile Phase A-ACN:MEOH:H2O:AA/ 5:10:85:1, Mobile phase B-ACN:MEOH:AA/ 40:60:1, Injection volume 20 μl, Flow rate 1 ml/min, Agilent RP-C8

Reverse phase HPLC based qualitative and quantitative sketching of *I. batatas* phenolics was done by comparing the retention time and UV spectra of reference compounds with those of the test sample, shown in Fig. 1. Rutin, gallic acid, catechin, caffeic acid, apigenin, myricetin, quercetin and kaempferol were identified and quantified in different extracts of IPT-EA, IPT-M, IPR-EA and IPR-M. Maximum amount of rutin was detected in IPT-EA (7.3 ± 1.12 μg/mg dry extract) while minimum quantity of apigenin (0.13 ± 0.020 μg/mg dry extract) was quantified in IPR-EA. The results are summarized in Table 2.

**Table 2:** Correlation of IC_50_ values of different antioxidant activities of *I. batatas* with total phenolic and total flavonoid contents

Antioxidant Activity	Correlation R^2^	
	TFC	TPC
DPPH radical scavenging activity	0.9382^*^	0.9732*
Nitric Oxide radical scavenging Activity	0.90455^*^	0.9754^*^
Hydroxyl radical scavenging activity	0.8487^ns^	0.9654^*^
Iron chelating assay	0.8139^ns^	0.7012^ns^
Phosphomolybdenum assay (TAC)	0.9837^**^	0.9975^**^
Reducing power assay (TRP)	0.9805^**^	0.9995^***^

Column with different superscripts are significantly correlated where ^*^ = *p* < 0.05^, **^ = *p <* 0.01^, ***^ = *p <* 0.001 and ^ns^ = *p* > 0.05 (non-significant), total flavonoid content (TFC), total phenolic content (TPC)

### In vitro antioxidant activities

*I. batatas* demonstrated dose dependent antioxidant activity in various in vitro antioxidant assays, including scavenging of DPPH, nitric oxide and hydroxyl (•OH) radicals as well as iron chelating competency. It also exhibited significant total reducing potential and total antioxidant capacity. *I. batatas* displayed antioxidant capacity in different assays in the subsequent order; nitric oxide scavenging > iron chelating ability > DPPH free radical scavenging > hydroxyl radical (•OH) scavenging (Additional file 1: Figure S1b). Furthermore, the total antioxidant capacity was found to be greater than its total reducing power potential as shown in Table 1. All the antioxidant assays were significantly correlated to TPC and TFC (Table 3).

**Table 3:** HPLC-DAD analysis of *I. batatas* extracts

Flavonoid/Phenolics	Signal wavelength	Quantity (μg/mg dry extract)			
		IPT-EA	IPT-M	IPR-EA	IPR-M
Rutin	257	7.3 ± 1.12^c^	0.75 ± 0.08^a^	4.5 ± 0.55^c^	3.7 ± 0.13^c^
Gallic acid	279	0.24 ± 0.080	nd	nd	Nd
Catechin	279	0.74 ± 0.04^a^	nd	nd	0.87 ± 0.09^a^
Caffeic acid	325	1.60 ± 0.25^a^	0.52 ± 0.07^a^	2.17 ± 0.26^b^	0.78 ± 0.11^a^
Apigenin	325	0.21 ± 0.040	0.18 ± 0.04	0.13 ± 0.020	0.31 ± 0.05
Myricetin	368	2.7 ± 0.14^b^	nd	01.01 ± .08^a^	0.26 ± 0.03
Quercetin	368	0.45 ± 0.05	nd	1.39 ± 0.12^a^	0.29 ± 0.06
Kaempferol	368	nd	nd	0.31 ± 0.040	Nd

Each value is presented as mean ± SD (*n* = 3)

*IPT-EA I. batatas* tuber-ethyl acetate extracts, *IPT-M I. batatas* tuber-methanol extract, *IPR-EA I. batatas* root-ethyl acetate extract, *IPR-M I. batatas* root-methanol extract

^a, b, c^ represents the significance of flavonoid/phenolics quantified and ^nd^ stands for not detected

### In vitro and in vivo toxicity investigation

**Fig. 2:**
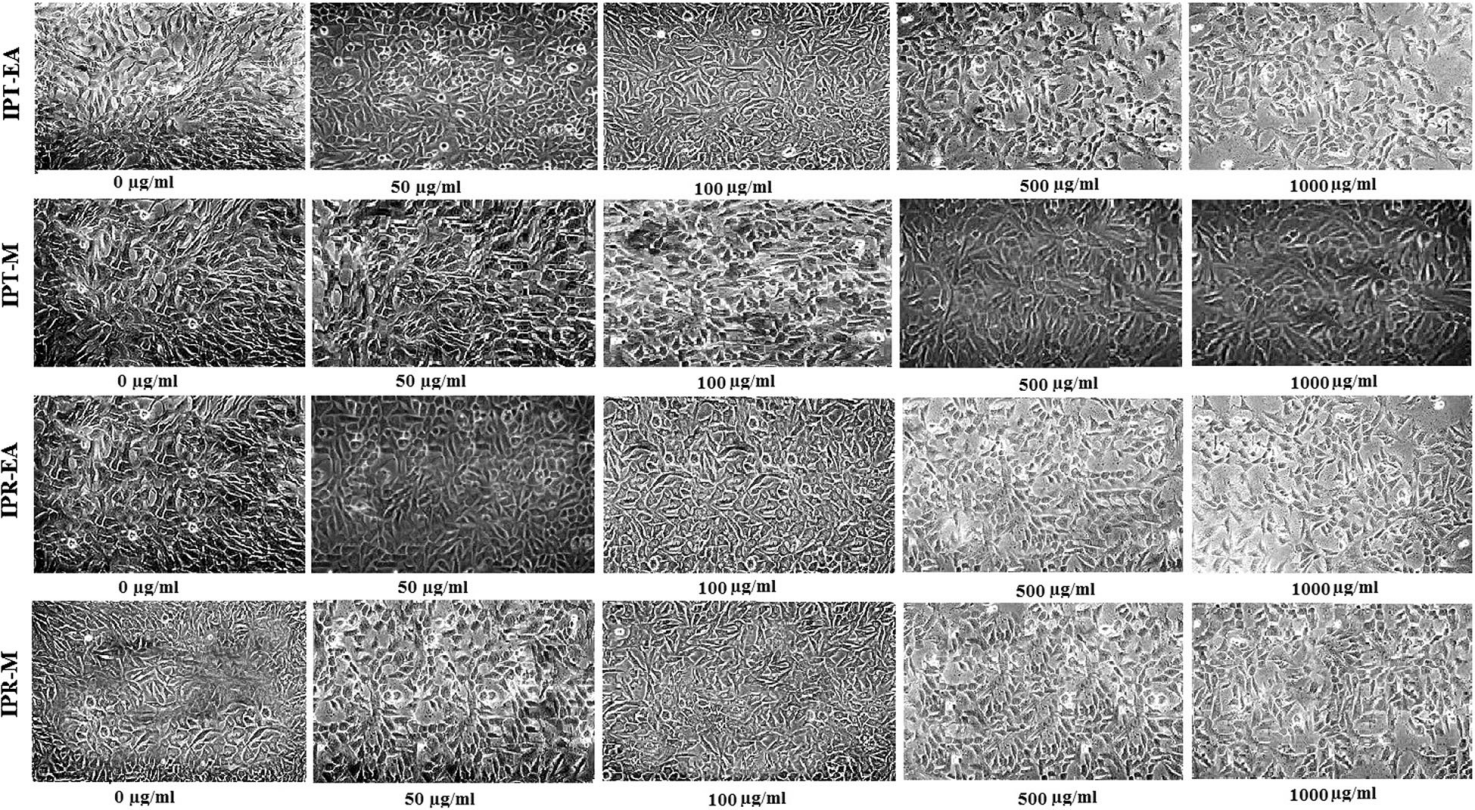
Effect of *Ipomoea batatas* on the cell viability of rabbit articular chondrocytes. Microscopic images of cells untreated or treated with indicated concentration of IPT-EA, IPT-M, IPR-EA and IPR-M. Results are mean ± SD of triplicate experiment

**Fig. 3:**
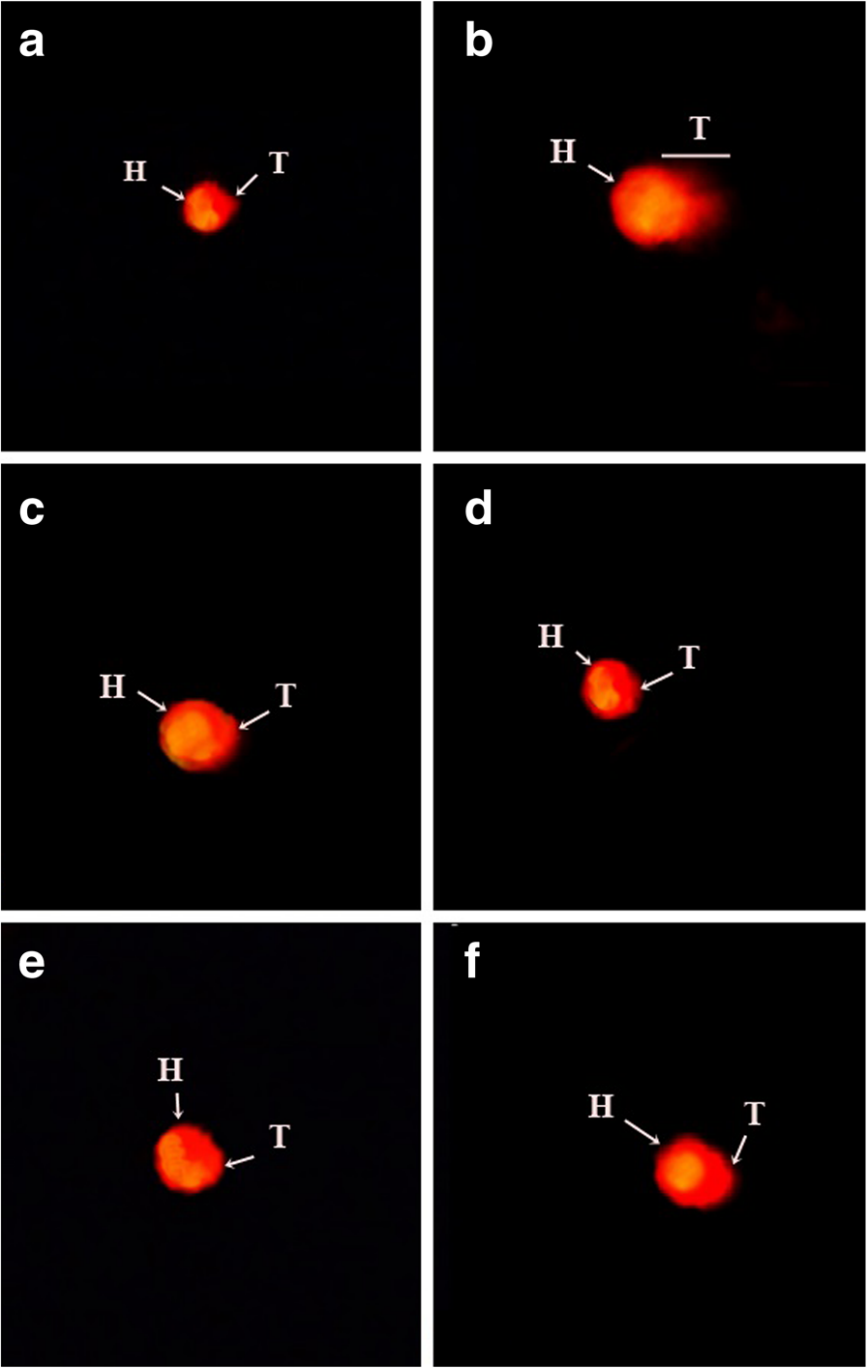
Genotoxicity evaluation of *I. batatas* on blood lymphocytes. **a** Vehicle control (1% DMSO) (**b**) Ethyl methane sulfonate (20 μg/ml) (**c**) IPT-EA (100 μg/ml) (**d**) IPR-EA (100 μg/ml) (**e**) IPR-M (100 μg/ml) and (**f**) IPT-M (100 μg/ml). “H” shows head of comet and “T” is the tail

In vitro results showed non-toxic nature of *I. batatas* on rabbit articular chondrocytes as evaluated by microscopic observations and MTT viability assay at various doses (0–1000 μg/ml) and the results are presented in Fig. 2 and Additional file 2: Figure S2b. Non-toxic nature of the *I. batatas* extracts was also confirmed by neutrophil genotoxicity assessment via comet assay as shown in Fig. 3 and Table 4. Further, results of acute toxicity showed that *I. batatas* did not exhibit any significant variation in behavioral pattern and signs of toxicity or death during the observation period of 2 weeks. The investigation was performed at the doses of 300–4000 mg/kg of rats. The IPT-EA, IPT-M, IPR-EA and IPR-M extracts were found safe up to the highest dose of 4000 mg/kg. So, *I. batatas* extracts were rendered non-toxic and safe for additional pharmacological testing within described range.

**Table 4:** Cytotoxicity assessment on blood lymphocytes by comet parameters

Sample	Comet length(μm)	Head length(μm)	Tail length(μm)	% DNA in head	% DNA in tail	Tail moment(μm)
IPT-EA	51.56 ± 3.01	44.32 ± 2.56	7.24 ± 0.32^b^	85.95 ± 3.7^ab^	14.04 ± 1.48^b^	0.15 ± 0.03^b^
IPT-M	56.63 ± 4.21	47.12 ± 1.98	9.51 ± 0.25^b^	83.20 ± 4.17^b^	16.79 ± 1.32^b^	0.21 ± 0.04^b^
IPR-EA	49.21 ± 3.65	43.21 ± 2.78	5.99 ± 0.22^a^	87.82 ± 3.52^a^	12.17 ± 0.89^a^	0.11 ± 0.02^a^
IPR-M	53.87 ± 2.89	44.94 ± 2.43	8.93 ± 0.45^b^	83.42 ± 3.30^b^	16.57 ± 1.69^b^	0.18 ± 0.04^b^
EMS	60.31 ± 3.91	43.12 ± 3.21	17.21 ± 1.5^c^	71.67 ± 4.31^c^	28.33 ± 1.91^c^	1.68 ± 0.32^c^
Vehicle Control	46.11 ± 3.14	40.32 ± 2.78	5.79 ± 0.34^a^	88.44 ± 4.67^a^	11.56 ± 3.01^a^	0.12 ± 0.02^a^

IPT-EA, IPT-M, IPR-EA and IPR-M were dosed at 100 μg/ml whereas EMS (Ethyl methane sulfonate) was given in 20 μg/ml concentration. Vehicle = 1% DMSO. Values are expressed as mean ± SD (*n* = 6). Means with letter “b” indicate significant difference from normal control, “a” and “c” from Ethyl methane sulfonate treated group according to Kruskal-Wallis test at *p* < 0.05

### In vitro antiinflammatory proficiency

**Fig. 4:**
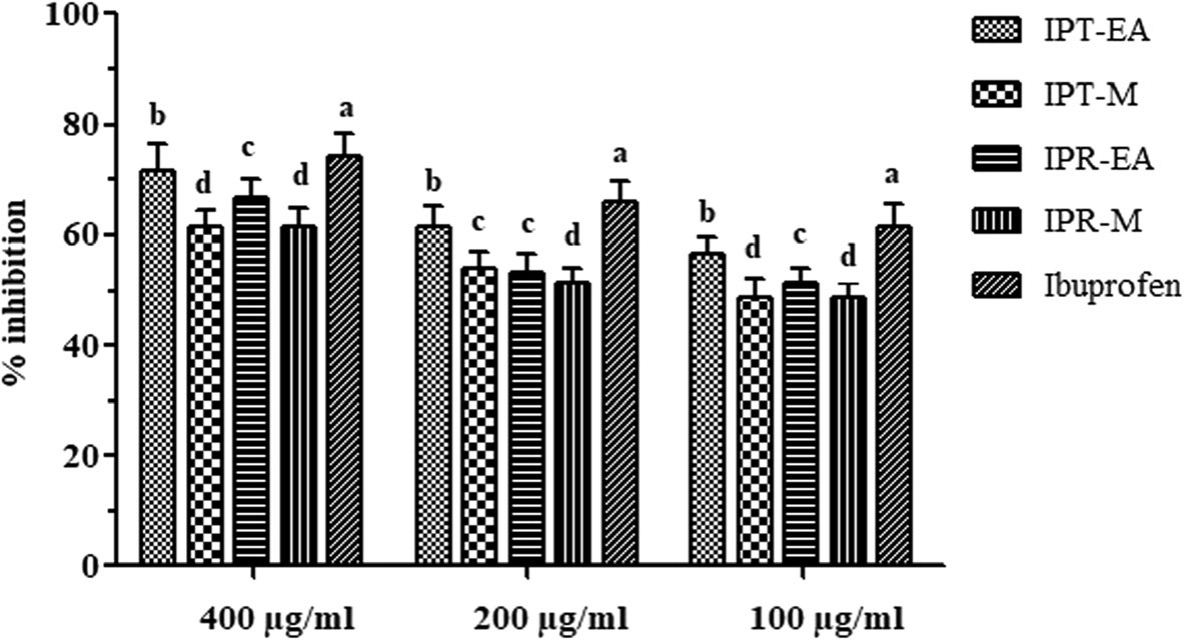
In vitro antiinflammatory assessment of *I. batatas* extracts via albumin denaturation. Each value is represented as mean ± SD (*n* = 3). Means with different superscript (a-d) letters in the column are significantly (*p* < 0.05) different from one another

For the evaluation of in vitro antiinflammatory potential of *I. batatas* extracts, inhibition of heat induced albumin denaturation assay was conducted. Maximum inhibition was exhibited by IPT-EA (76.92 ± 3.07%) followed by IPR-EA (71.79 ± 4.87%), IPR-M (66.66 ± 3.61%) and IPT-M (66.66 ± 2.76%) in comparison to the standard, ibuprofen (79.48 ± 4.71%) at the concentration of 500 μg/ml. The results are summarized in Fig. 4.

### Effect on carrageenan-induced inflammation in rats

Carrageenan-induced rat paw edema model (inflammatory model) was used to assess the antiinflammatory activity of the sample. The results showed that *I. batatas* have significant antiinflammatory activity compared to the control model. IPR-EA showed maximum inhibition of inflammation (79.11 ± 5.47%) at 6 h. It was followed by IPT-EA (74.20 ± 5.14%), IPR-M (68.67 ± 5.32%) and IPT-M (63.02 ± 4.21%) in comparison to ibuprofen (85.25 ± 6.98%). Experimental results are presented in Table 5.

**Table 5:** Effect of *I. batatas* on carrageenan-induced paw edema in rat

Groups	Edema volume (ml) Percent edema inhibition				
	1st h	3rd h	6th h	12th h	24th h
IPT-EA(300 mg/kg)	0.28	0.39	0.21	0.285	0.386
	24.52 ± 2.81^c^	38.58 ± 2.67^bc^	74.20 ± 5.14^c^	70.97 ± 5.75^c^	66.49 ± 4.30^c^
IPT-M (300 mg/kg)	0.315	0.487	0.301	0.39	0.542
	15.09 ± 1.43^e^	23.30 ± 1.70^d^	63.02 ± 4.21^e^	60.28 ± 5.13^e^	52.95 ± 4.13^e^
IPR-EA (300 mg/kg)	0.26	0.364	0.17	0.24	0.34
	29.91 ± 2.91^b^	42.67 ± 3.71^b^	79.11 ± 5.47^b^	75.56 ± 6.06^b^	70.48 ± 4.61^b^
IPR-M (300 mg/kg)	0.3	0.41	0.255	0.335	0.463
	19.13 ± 1.72^d^	35.43 ± 3.07^c^	68.67 ± 5.32^d^	65.88 ± 5.94^d^	59.80 ± 3.90^d^
Ibuprofen (10 mg/kg)	0.22	0.26	0.12	0.17	0.26
	40.70 ± 3.08^a^	59.05 ± 4.51^a^	85.25 ± 6.98^a^	82.68 ± 6.39^a^	77.43 ± 6.05^a^
Carrageenan Control	0.371	0.635	0.814	0.982	1.152
Saline Control	0.256 ± 0.05	0.301 ± 0.04	0.2 ± 0.03	0.14 ± 0.03	0.11 ± 0.04

Data values shown represent mean ± SD (*n* = 6). Means with different superscript (^a-e^) letters in the column are significantly (*p* < 0.05) different from one another

### Croton oil-induced inflammation inhibition

Croton oil-induced ear edema and anal edema model (inflammatory model) were used to further assess the antiinflammatory activity of the *I. batatas.* The results showed that *I. batatas* extracts have significant antiinflammatory activity compared to the control model. IPR-EA showed maximum inhibition of ear inflammation (72.01 ± 7.80%) followed by IPT-EA (62.94 ± 4.12%), IPR-M (60.03 ± 4.22%) and IPT-M (52.58 ± 3.45%) in comparison to ibuprofen (80.58 ± 5.03%). Moreover, IPR-EA showed maximal inhibition of anal inflammation (70.80 ± 4.94%) followed by IPT-EA (63.04 ± 4.17%), IPR-M (54.53 ± 3.80%) and IPT-M (51.75 ± 3.79%) in comparison to ibuprofen (82.50 ± 6.21%). Experimental results are presented in Table 6.

**Table 6:** Effect of *I. batatas* on croton oil-induced ear and anal edema in rat

Groups	Weight of left ear (mg)	Weight of right ear (mg)	Edema (∆ mg)	% reduction of inflammation	Anus size (mm)	Edema (mm)	% reduction of inflammation
IPT-EA	91.44 ± 1.7	93.73 ± 1.5	2.29 ± 0.20	62.94 ± 4.1^c^	39.13 ± 1.65	4.52 ± 0.21	63.04 ± 4.17^c^
IPT-M	87.3 ± 1.30	90.23 ± 1.2	2.93 ± 0.25	52.58 ± 3.4^d^	40.81 ± 1.87	5.90 ± 0.32	51.75 ± 3.79^d^
IPR-EA	86.34 ± 2.1	88.07 ± 1.8	1.73 ± 0.15	72.01 ± 7.8^b^	38.18 ± 1.43	3.57 ± 0.29	70.80 ± 4.94^b^
IPR-M	83.8 ± 1.02	86.27 ± 1.2	2.47 ± 0.11	60.03 ± 4.2^c^	40.17 ± 1.87	5.56 ± 0.38	54.53 ± 3.80^d^
Ibuprofen	84.7 ± 1.67	85.9 ± 2.01	1.20 ± 0.10	80.5 ± 5.03^a^	36.75 ± 1.01	2.14 ± 0.12	82.50 ± 6.21^a^
Croton oil	93.4 ± 1.10	99.58 ± 2.1	6.18 ± 0.74		46.84 ± 1.21	12.23 ± 0.8	
Normal	80.04 ± 1.4	80.0 ± 2.02			34.61 ± 0.90		

Data values shown represent mean ± SD (*n* = 6). Means with different superscript (^a-d^) letters in the column are significantly (*p* < 0.05) different from one another

### In vivo antiarthritic activity on CFA-induced arthritic rats

#### Effect on paw volume in treatment mode

**Fig. 5:**
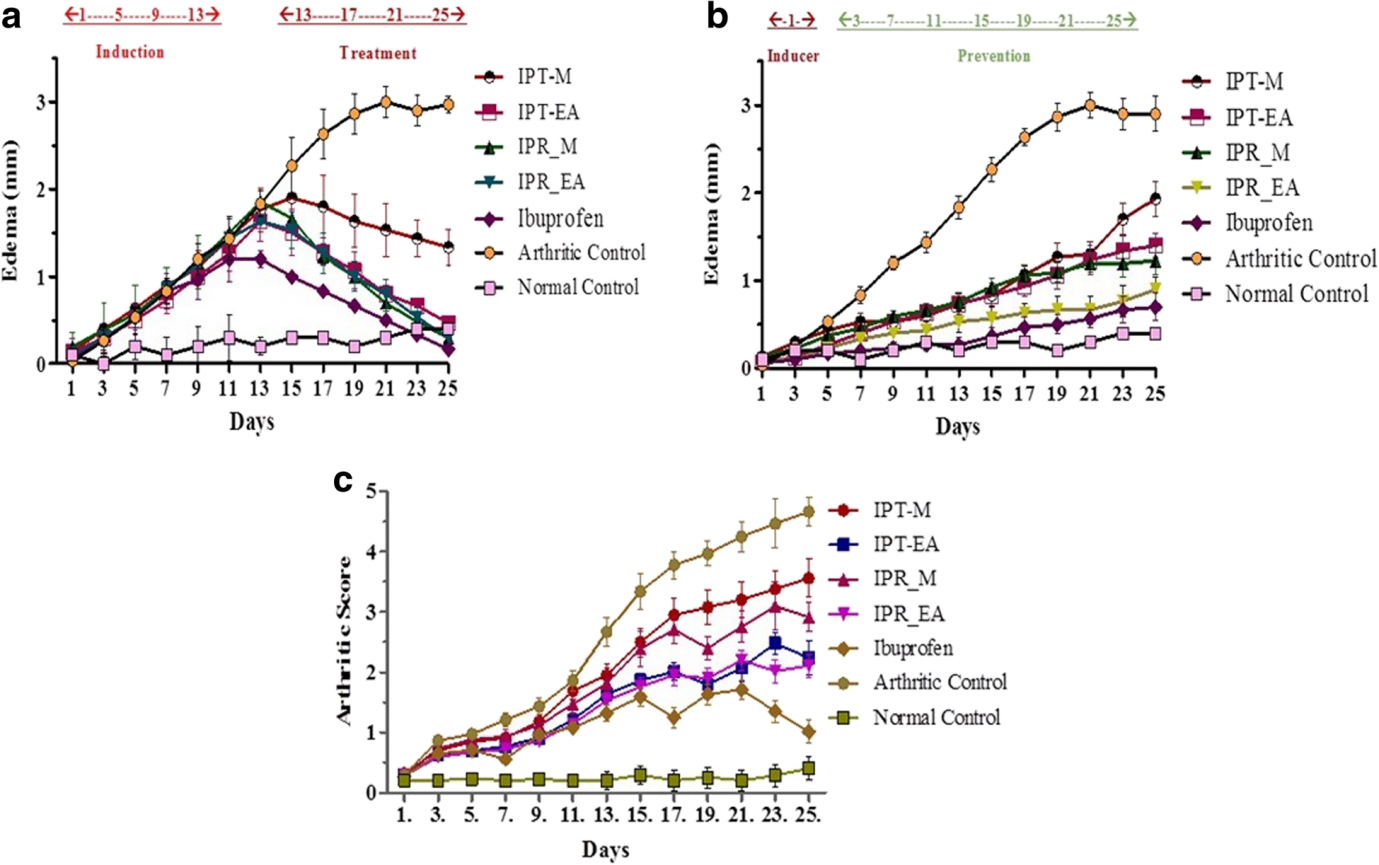
Assessment of arthritic edema and arthritic score. Data is presented as the mean arthritic score ± SEM (*n* = 6/group). **a** Treatment mode of studies where the induction was done till day 13 and then dose was started. Edema in arthritic control group increased with the passage of time. IPR-EA and IPR-M have significantly decreased arthritic edema after 15th day in comparison to ibuprofen. **b** Preventive mode of study where dose was given after inducer at day 1. Arthritic control group has not prevented the induction of the disease while IPR-EA and IPR-M have significantly prevented arthritic edema in comparison to ibuprofen. **c** Arthritic score where arthritic control group has higher arthritic score compared to normal and other groups. The ibuprofen treated animals showed significant decrease in arthritic score after first week onwards during the experiment

Antiarthritic efficacy of *I. batatas* was evaluated by measuring the paw volume. Swelling of paw is the most critical contributing factor in the evaluation of inflammation severity and also shows the curative efficacy of the antiarthritic drugs. Treatment with IPT-EA, IPT-M, IPR-EA and IPR-M extracts (300 mg/kg) and ibuprofen (10 mg/kg) have exhibited significant reduction in paw edema from the 15th to 25th day (*p* < 0.05) as compared to arthritic control group (Fig. 5a).

#### Effect on paw volume in preventive mode

After injecting CFA to induce arthritis, rat groups were given IPT-EA, IPT-M, IPR-EA and IPR-M extracts (300 mg/kg) and ibuprofen (10 mg/kg) straightaway from day 1st and the prevention of arthritis has been assessed by measuring the hind paw volume. *I. batatas* and -ibuprofen exhibited maximal prevention from arthritic inflammation of paw (Fig. 5b). Arthritic scoring was also done according to the said procedure and maximum recovery from disease was observed in ibuprofen, which lowered the swelling from 17th day of dosing and onward followed by IPR-EA, IPT-EA, IPR-M and IPT-M (Fig. 5c).

#### Effect on arthritis-induced physical changes

**Fig. 6:**
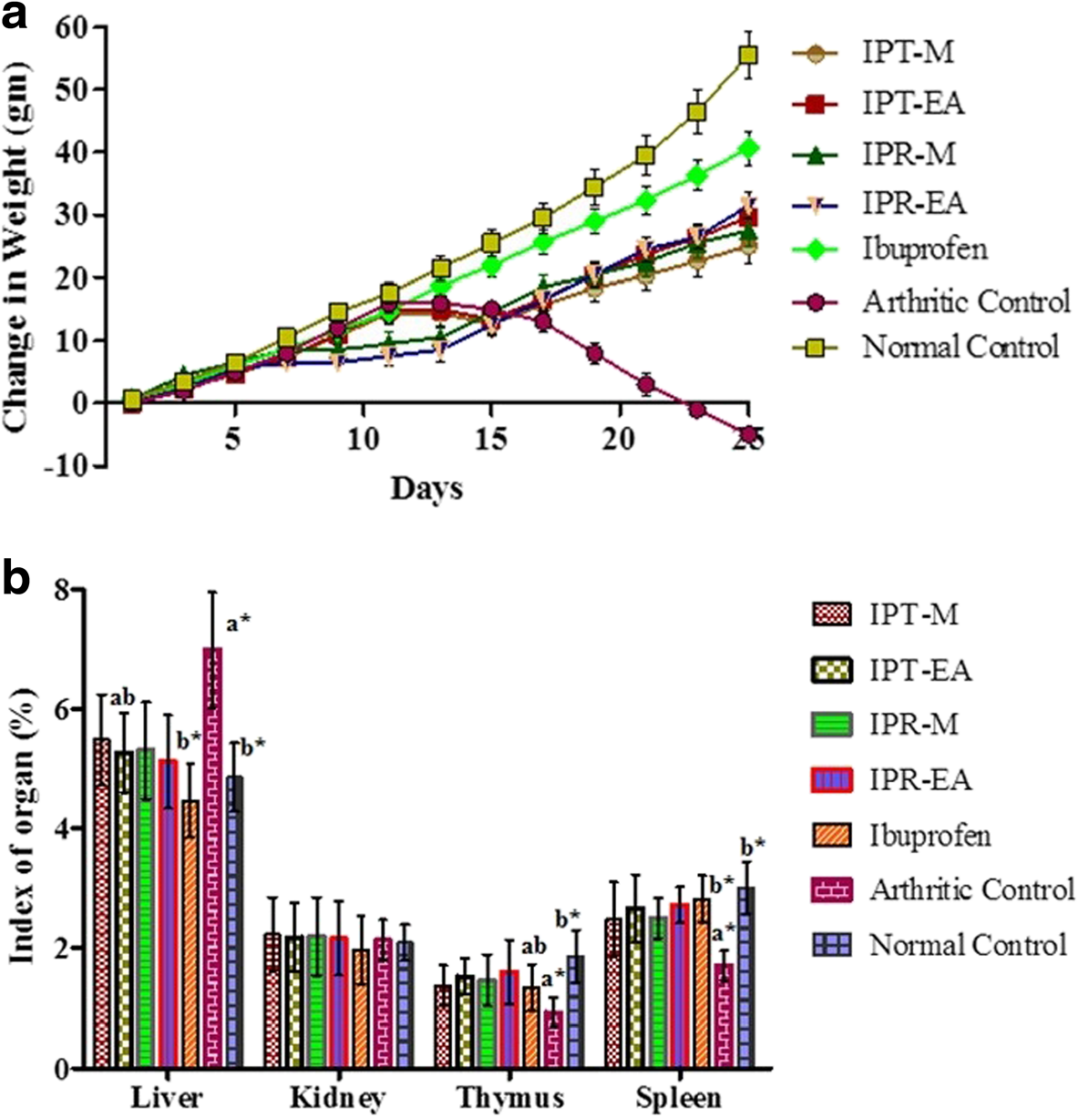
Assessment of the change in body weight and organ index in experimental animals. **a** Variation in mean body weight over indicated period of time. **b** Normal and arthritic altered organs index. Each value represents the mean ± SD (*n* = 6/group). Differences were considered significant at the level of * *p* < 0.05

In the current study, we have studied the in vivo efficacy of *I. batatas* on CFA-induced arthritic physical changes. At the final day of experiment, arthritic rats (− 5.00 ± 1.28 g, *p* < 0.05) were found to have significantly lower body weight change compared to the normal rats (55.5 ± 3.7 g) as presented in the Fig. 6a. *I. batatas* treated rats exhibited an increase in the body weight as IPR-EA (31.5 ± 2.06 g) followed by IPT-EA (29.67 ± 1.98 g), IPR-M (27.51 ± 2.7 g) and IPT-M (25.01 ± 2.7 g) while ibuprofen treated rats showed an increase in body weight of 40.66 ± 2.7 g (Fig. 6a). Furthermore, relative organ weights were measured, which are presented as an index of the organ. As arthritis affects the key organs of an organism; therefore, in the present study arthritic-induced effects on major organs such as liver, kidney, thymus and spleen were also determined. After the completion of treatment, arthritic rats were found to have a significantly higher liver index, a decreased thymus and spleen index while the effect on the kidney was statistically insignificant compared to the normal rats as shown in the Fig. 6b. Administration of IPR-EA restored the liver (12.96%), thymus (26.20%) and spleen (17.54%) followed by IPT-M (8.43, 18.19 and 11.58%, respectively), IPT-EA (9.25, 21.39 and 16.88%, respectively) and IPR-M (5.34, 13.91 and 9.62%, respectively) at the dose of 300 mg/kg compared to the arthritic control. On the other hand, ibuprofen exposed animals showed 10.3, 17.7 and 21.68% recovery in same organs, respectively.

#### Effect on histology of normal and inflamed joint

**Fig. 7:**
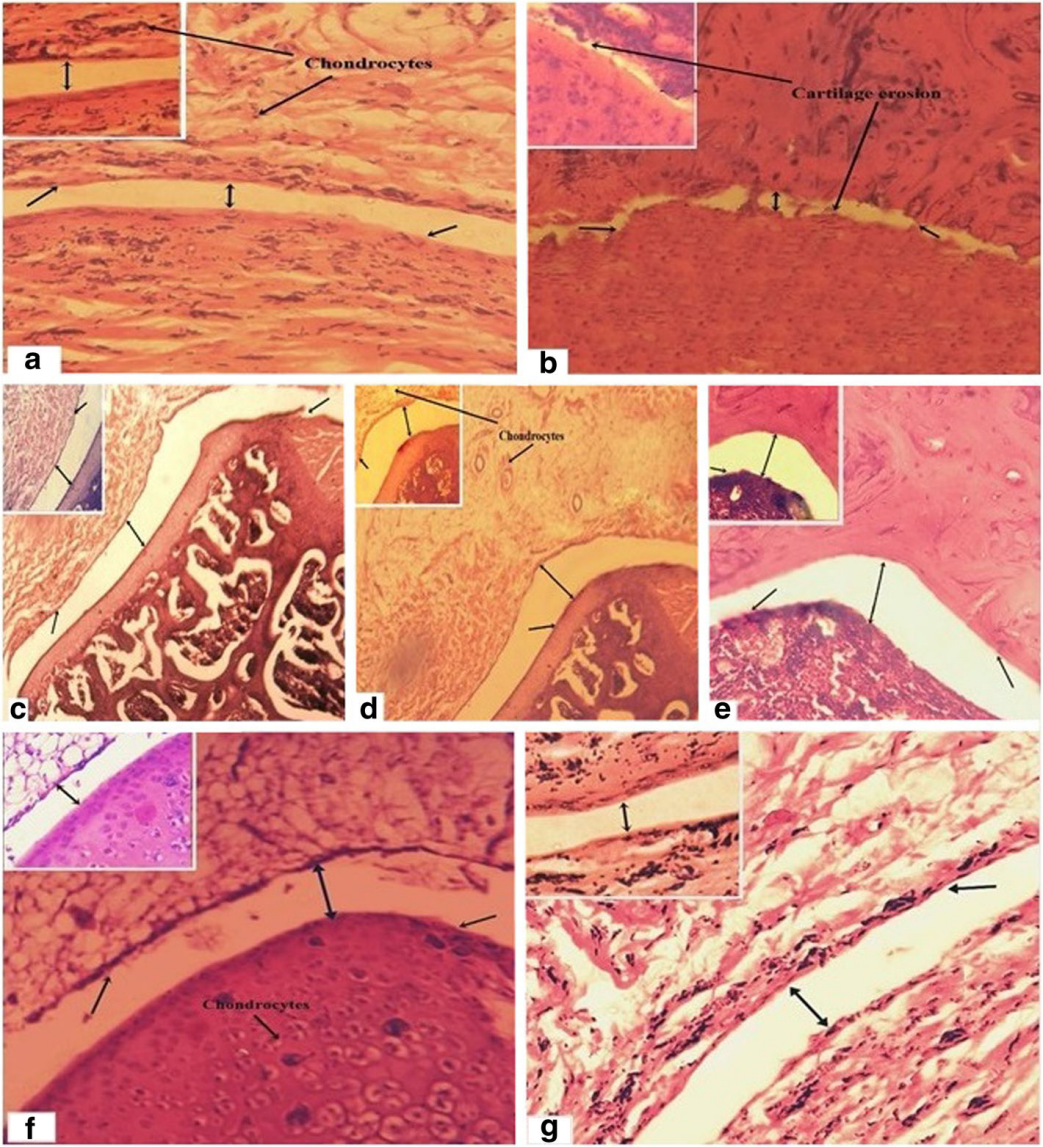
Investigation of histological architecture (**a**) normal rat; (**b**) arthritic rat; (**c**) IPR-EA 300 mg/kg; (**d**) IPT-EA 300 mg/kg; (**e**) IPR-M 300 mg/kg; (**f**) IPT-M 300 mg/kg; (**g**) ibuprofen 10 mg/kg. The black double arrow shows variation in joint spaces among different groups, while single arrows represent the chondrocytes or cartilage erosion

The histological evaluation revealed that arthritic rats have severe edema, inflammatory cells infiltration and abnormal joint architecture with increased erosion, cartilage destruction and decreased joint space. However, IPR-EA, IPT-M, IPR-EA, IPR-M extracts and standard drug treated groups exhibited protective effects on the altered histology and joint architecture (Fig. 7).

#### Arthritis-induced hematological variations

The results of the arthritis-induced hematological variations are presented in Table 7. It was observed from the results that arthritis progression altered the hematological parameters such as decrease in hemoglobin RBCs, platelets, whereas augmented levels of ESR and WBCs in arthritic animals. Administration of the *I. batatas* extracts significantly restored these altered hematological parameters as shown in the Table 7.

**Table 7:** The haematological investigation of normal control and arthritic rats

Groups	RBCs (× 10^6^)/μl	WBCs (×10^3^) /μl	Platelets (×10^3^) /μl	Hb (g/dl)	ESR (mm/h)
IPT-EA	5.81 ± 0.019^b^	3.89 ± 0.011^ab^	558 ± 02.811^ab^	8.03 ± 0.47^b^	4.98 ± 0.053^b^
IPT-M	5.40 ± 0.013^b^	4.67 ± 0.012^b^	498 ± 03.2120^c^	7.81 ± 0.54^c^	5.49 ± 0.089^c^
IPR-EA	6.01 ± 0.012^ab^	3.72 ± 0.014^ab^	571 ± 02.710^ab^	8.26 ± 0.71^b^	4.83 ± 0.072^b^
IPR-M	5.62 ± 0.071^b^	4.15 ± 0.017^b^	535.10 ± 1.91^b^	7.72 ± 0.25^c^	5.13 ± 0.10^bc^
Ibuprofen	5.44 ± 0.032^b^	6.31 ± 0.020^c^	394.5 ± 02.29^d^	7.84 ± 0.70^c^	4.96 ± 0.023^b^
Arthritic Control	4.14 ± 0.025^c^	7.02 ± 0.035^d^	346.67 ± 2.51^e^	6.31 ± 0.12^d^	9.44 ± 0.061^d^
Normal	6.78 ± 0.078^a^	3.51 ± 0.045^a^	594.81 ± 4.58^a^	10.33 ± 0.9^a^	4.35 ± 0.045^a^

Results are presented as mean ± SD (*n* = 6). Means with different superscript (^a-e^) letters in the column are significantly (*p* < 0.05) different from one another

#### Enzymatic and biochemical regulation

Results showed that *I. batatas* extracts were effective in restoring the stress induced altered enzyme levels and biochemical parameters such as ALT, AST, ALP, urea, creatinine, albumin, and decreased levels of testosterone, bilirubin in arthritic rats. Administration of the *I. batatas* extracts significantly recovered the biochemical and enzymatic profile and detailed results are presented in Table 8.

**Table 8:** Enzymatic and biochemical investigation of control and arthritic rats

Groups	ALT (U/L)	AST (U/L)	ALP (U/L)	Urea (mg/dl)	Creatinine(mg/dl)	Bilirubin (mg/ml)	Albumin (mg/dl)	Testosterone (ng/ml)
IPT-EA	60.21 ± 2.7^c^	56.3 ± 2.4^c^	156.4 ± 3.8^d^	61.12 ± 2.8^c^	1.63 ± 0.1^d^	6.03 ± 0.1^d^	5.58 ± 0.19^c^	5.87 ± 0.4^b^
IPT-M	73.16 ± 2.9^b^	67.2 ± 2.1^b^	184.3 ± 5.8^b^	66.71 ± 2.3^b^	1.8 ± 0.21^b^	5.8 ± 0.12^f^	5.21 ± 0.30^b^	5.46 ± 0.3^d^
IPR-EA	54.77 ± 2.8^d^	47.2 ± 2.5^d^	141.3 ± 4.3^e^	57.07 ± 1.8^d^	1.56 ± 0.12^e^	6.1 ± 0.07^c^	5.8 ± 0.12^cd^	6.37 ± 0.8^a^
IPR-M	69.2 ± 2.4^bc^	64.3 ± 2.9^bc^	172.8 ± 5.4^c^	63.3 ± 2.4^bc^	1.76 ± 0.11^c^	5.9 ± 0.11^e^	5.3 ± 0.24^bc^	5.7 ± 0.45^c^
Ibuprofen	45.44 ± 2.1^e^	39.9 ± 1.3^e^	123.3 ± 2.9^f^	54.1 ± 1.9^de^	1.48 ± 0.11^f^	6.2 ± 0.08^b^	5.91 ± 0.23^d^	5.9 ± 0.38^b^
Arthritic Control	93.37 ± 2.4^a^	103.9 ± 2.1^a^	334.1 ± 7.1^a^	71.3 ± 2.34^a^	2.54 ± 0.22^a^	5.6 ± 0.21^g^	2.71 ± 0.14^a^	4.37 ± 0.4^e^
Normal	39.00 ± 0.5^e^	26.7 ± 0.6^f^	115.2 ± 2.1^g^	51.3 ± 1.89^e^	1.34 ± 0.12^g^	6.4 ± 0.23^a^	6.11 ± 0.28^e^	6.6 ± 0.67^a^

Data is mean ± SD (*n* = 6). Means with different superscript (^a-g^) letters in the column are significantly (*p* < 0.05) different from one another

#### Effect on antioxidant enzymes

**Fig. 8:**
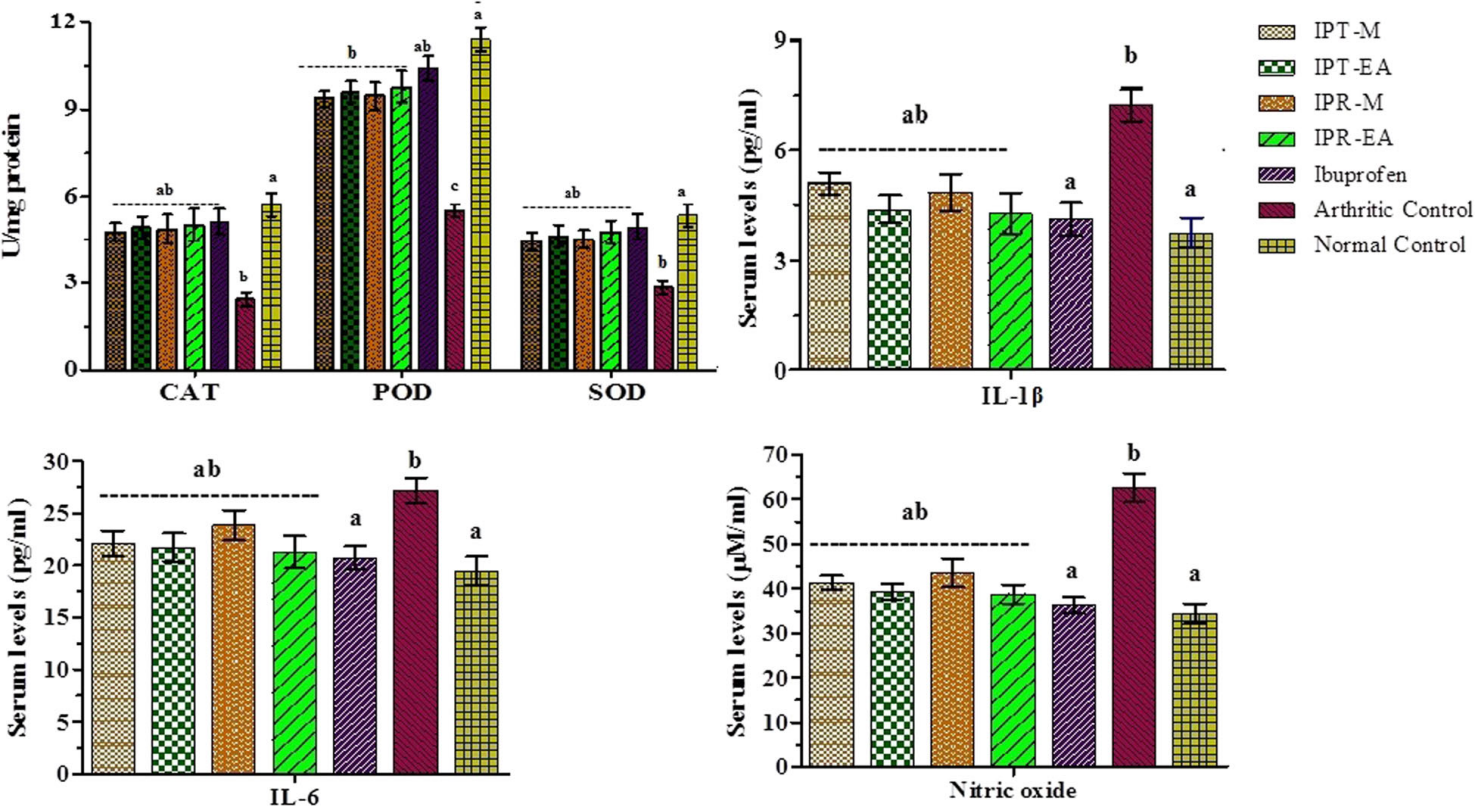
Effect of *Ipomoea batatas* extracts on CFA-induced arthritis stimulated endogenous antioxidant enzymes, IL-1β, IL-6 and NO levels in experimental animals. Catalase (CAT), Peroxidase (POD), Superoxide dismutase (SOD), Interleukin (IL) and Nitric oxide (NO). Data is presented as mean ± SD (*n* = 6). Differences were considered significant at the level of * *p* < 0.05

Arthritic condition causes the induction of oxidative stress and endogenous antioxidant enzymes are critical system to counteract such oxidative damages. A significant reduction in expression of antioxidant enzymes such as CAT, POD, and SOD was observed in the arthritic group by 57.02, 51.88 and 47.01%, respectively in comparison to normal control. Compared to arthritic group, ibuprofen treated animals established a reduction in expression of 10.17, 8.74 and 7.49%, respectively. In case of *I. batatas*, IPR-EA altered the levels of CAT, POD and SOD by 12.11, 14.43 and 10.86%, respectively while IPT-M altered maximally by 16.32, 18.11 and 16.85%, respectively. Results are presented in the Fig. 8.

#### Effect on interleukins and NO levels

Levels of interleukins and NO are supposed to increase in arthritic condition, as these macromolecules have been shown to modulate extracellular matrix turnover, accelerate the degradation of cartilage and induce chondrocyte apoptosis in the development of OA. Highest levels of IL-1β (7.23 ± 0.43 pg/ml), IL-6 (27.16 ± 1.22 pg/ml) and NO (62.56 ± 3.23 μM/ml) were shown by arthritic control in comparison to IPR-EA (4.28 ± 0.55 pg/ml, 21.28 ± 1.56 pg/ml and 38.73 ± 2.11 μM/ml) and ibuprofen (4.11 ± 0.44 pg/ml, 20.72 ± 1.14 pg/ml and 36.22 ± 1.78 μM/ml). Results are presented in the Fig. 8.

## Discussion

Immune system is both a blessing as well as a curse in certain situations. It may bring about certain incurable diseases due to some serious hypersensitive or allergic reactions causing various complications of intense severity like serum sickness, myasthenia gravis, pernicious anemia and reactive arthritis with unknown etiology [43]. RA is one of the most common autoimmune inflammatory conditions of indefinite etiology well-appointed with symmetric erosive synovitis and in fewer cases extra-articular engrossment [44]. Cytokines are the key factors with job to regulate a variety of inflammations, which are involved in the onset and pathogenesis of rheumatoid arthritis. Tendered joints with degrees of arthritic swellings, definitely creates a situation of imbalance between pro and antiinflammatory cytokines, which is an open invitation to autoimmune reaction ultimately commencing chronic inflammation and joint injury [45]. Cartilage abrasion, tissue destruction and hyperalgesic situations due to inflammation are mainly due to cyclooxygenase, arachidonic acid and lipooxygenase triggered local inflammatory mediators like prostaglandins, leukotrienes, thromboxane A_2_ and prostacyclin [46]. In early acute phase, arthritis comprises pain of severe degrees, lack of mobility, cessation of body weight gain and inflammation and swelling of hind and fore paw joints while in late acute phase (12+ days) rats are unable to move because of high level joint inflammations [2]. Cartilage can bear a high degree of stress and load due to its extracellular matrix constituting proteoglycan (PG) and collagen helix fibers [47]. Cartilage tissues exclusively lack vascularization, have least number of stem cells and measured turnover of collagen, which clearly depict its limited interior repair extent and ability. Yet extrinsic mechanism of mesenchymal stromal cells of connective tissues play some role in cartilage repairing. When ROS level exceeds the biological concentrations, it plays role in amplification and aggressing RA [48]. Histological slides clearly depict these worst conditions in disease controls. The characteristic ability of medicinal plants to cure a number of diseases due to existence of versatile compounds of therapeutic worth has heightened their reputation and is aiding physicians to fight confidently against the upcoming disorders [49].

In the present study, phytochemical screening of IPT-EA, IPT-M, IPR-EA and IPR-M yielded TPC and TFC, justifying the medicinal inference of the food plant. HPLC-DAD quantified significant amounts of rutin, gallic acid, caffeic acid, catechin, apigenin, myricetin and quercetin that is additional clue for the medicinal propensity of the plant. Rutin, gallic acid and quercetin are acknowledged and well alleged secondary metabolites of plants with admirable role in hepato-protection, neurological disorders and apoptosis as well as have antiinflammatory and antioxidant potential [10]. Similarly, complementary antioxidant results in the plant extracts are due to these proficient polyphenols. These polyphenols added massive abilities to *I. batatas* extracts to scavenge high counts of oxidants.

*I. batatas* extracts were evaluated for the chondrocyte toxicity and viability testing and genotoxicity via lymphocytic electrophoresis. Most of the exposed chondrocytes remained viable at various increasing doses of *I. batatas* extracts. For the evaluation of safety parameters at molecular level, assessment of lymphocyte genotoxicity via comet assay has been conducted. Size of comet head and concentration of the DNA in that region was proof of geno-protective aptitude of food plant. Comet tail was much smaller than that of standard geno-toxic drug ethyl methane sulfonate proving DNA protecting aptitude of the extracts. The non-toxic nature of plant sanctioned its safety and suitability for in vivo usage. Outcomes of acute toxicity added to the safety of extracts as high doses up to 4000 mg/kg body weight had no deleterious effects in rats.

A physical or chemical stress like use of heat, strong acid or base, an organic solvent or concentrated inorganic salt that cause loss of tertiary and secondary structure of protein is termed as protein denaturation. This denaturation of protein is the leading cause of inflammation [50]. To evaluate mechanistic antiinflammatory activity, competence of the plant extract to inhibit protein denaturation was appraised. It was effective in inhibiting heat induced albumin denaturation. Maximum inhibition of 76.92 ± 3.07% was observed at the concentration of 500 μg/ml in comparison to the standard drug ibuprofen (79.48 ± 4.71%). Administration of *I. batatas* did not exhibit any physiological and behavioral adverse effect or mortality in rat model; hence, recommended safer for further investigation in test animals. Among the various models of antiinflammatory activity assessment, carrageenan-induced inflammatory model is a typical model used to investigate the activity of trial drugs [30]. Development of edema with carrageenan is a biphasic model, which involves the contribution of vascular and inflammatory mediators. Initial phase (0–1 h) of edema is attributed by the release of histamine, 5-hydroxytryptamine and bradykinin and is not repressed by the use of NSAIDs such as ibuprofen or aspirin. During the 2nd phase (1–6 h) of edema development, elevated levels of prostaglandins and inducible cyclooxygenase (COX-2) have been demonstrated in hind paw edema of rat. The inhibition of inflammation during the early and the late phase of edema with *I. batatas* extracts suggest that they have reduced the antiinflammatory activity by constraining the release of mediators of inflammation. Presence of polyphenols in the extracts has contributed towards the antiinflammatory activity by inhibiting the infiltration of neutrophils, suppression of IL-1β, IL-6 and NO [51].

Histamine is released in response to allergic reactions and causes skin, nose, throat and lung irritation. During inflammation, it plays its role through vasodilation, edema, increased vascular permeability and recruitment of eosinophil. It regulates the leukocyte function and migration, proliferation of T cells, B cell differentiation and release of lysosomal enzymes in neutrophils [52]. The antiinflammatory activity of any extract/compound can be assessed by the croton oil-induced ear and anus edema. Endothelial nitric oxide synthase (eNOS) plays a pivotal role during the histamine-induced paw edema. Thus, lowering down the levels of NO may aid in the reduction of inflammation as NO is directly accounted for the macromolecular gush induced by histamine inflammation [53]. In this study, *I. batatas* extracts appreciably inhibited the croton oil-induced inflammation in rat ear and anus suggesting that the antiinflammatory efficacy observed is because of the stabilization of mast cell membranes, lowering down the levels of NO synthesis and histamine inhibition. Earlier Sajid et al. [50] has narrated the similar histamine based inflammatory findings.

**Fig. 9:**
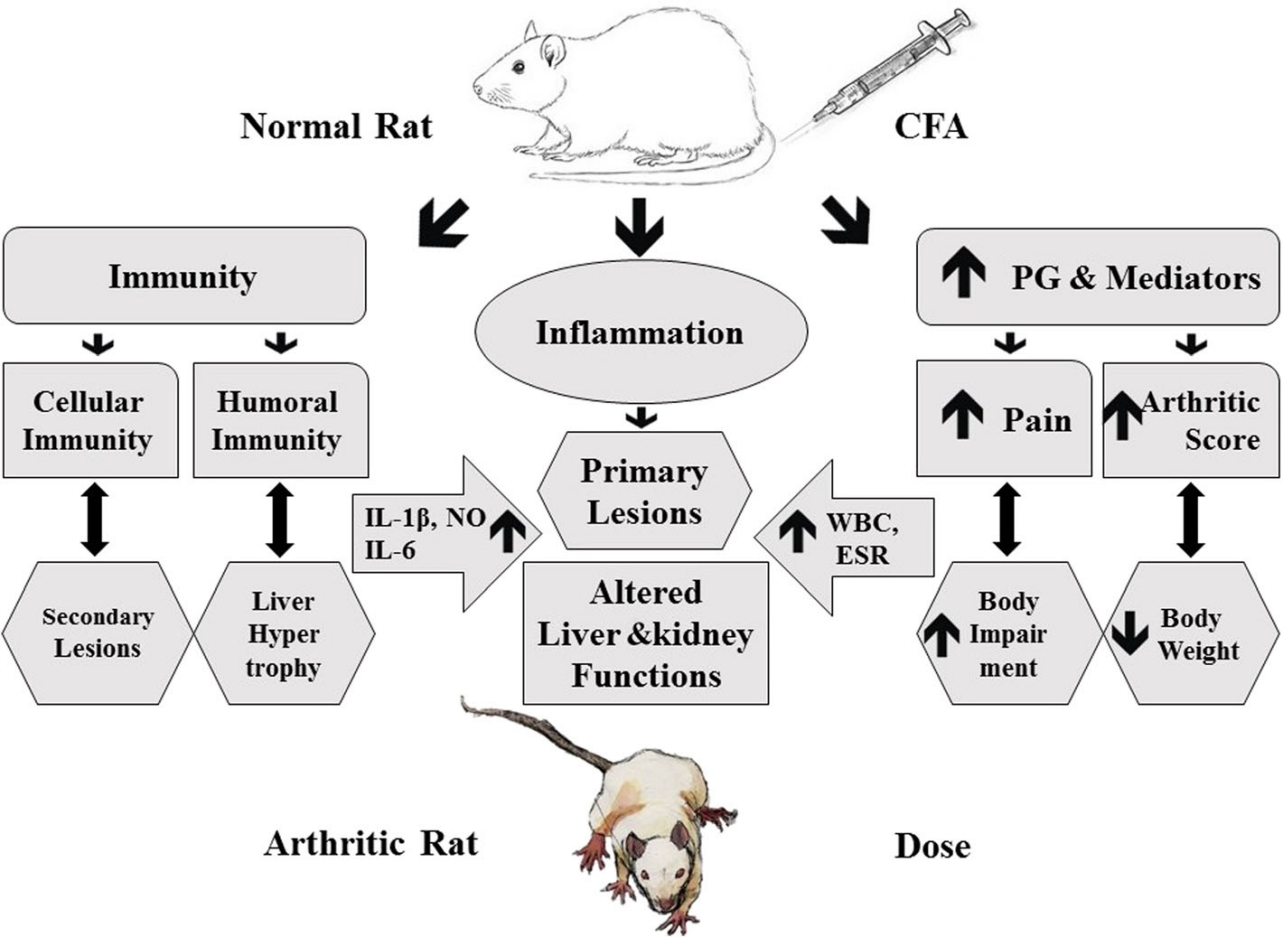
Proposed mechanism and pathway of CFA-induced arthritis showing onset of disease and various local and systemic variations in the body of experimental rat. The pathways have been reproduced by Muhammad Majid (1st author) from already published paper [56] under copyright licence no: 4101750876963 dated 04/05/2017

Adjuvant-induced arthritic rat model has extensively been used as classical animal model sharing several characteristic features of human rheumatoid arthritis patients, such as immunology and histology [54]. Adjuvant-induction results in activation of the cell-mediated immune response and stimulates immunoglobulin production in organisms, which results in the induction of primary and secondary chronic arthritis [55]. A large amount of pro-inflammatory cytokines are produced in adjuvant arthritis that may cause inflammation at different sites, also known as polyarthritis. Pro-inflammatory cytokines and interleukins are involved in osteoclast differentiation, inflammation and bone erosion [50]. The scheme of CFA-induced arthritis has been summarized in Fig. 9 [56].

In the present study, extracts of *I. batatas* tuber and root have significantly reduced the extent and level of inflammation in the late phase (fourth week) of the disease in comparison to the arthritic control with progressive inflammation and lesions. Inhibition of TNF-α and IL-1β, IL-2, IL-6 and NO mainly contributes in the eradication of inflammations as these macromolecules are purely responsible for the synthesis of metallo-proteinases and proliferation of synovial cells resulting in cartilage degradation [57]. The inhibition of IL-1β, IL-6 and NO proves *I. batatas* as an antiarthritic agent as not only levels of inflammation have been reduced as obvious in arthritic score, but cartilage erosion is also minimized (histology). Significant inhibition of inflammation in treatment mode of the study is possibly due to the suppression of these over expressing cytokines causing damages to the joint cartilage and synovial cell proliferation. Similarly, the plant extracts exhibited significant inhibition of the onset of disease in preventive mode of study, which justifies its antiarthritic capabilities as they subdued the levels of pro-inflammatory cytokines. Previous studies reported that polyphenols like caffeic acid [58], rutin and quercetin [59], gallic acid [60], kaempferol [61], catechin [62] and apigenin [63] are good antiinflammatory compounds that decrease the inflammatory reaction by blocking neutrophil transfer associated with the reduction in TNF-α, IL-1β levels and oxidative stress [51]. Additionally our study is in good correlation with the phenolic contents quantified. The extracts with high TPC and TFC showed best results in multiple experiments; hence, authenticating the antiinflammatory role of polyphenols. Our findings are in consensus with the outcomes of Younis et al. [1].

In the current investigation, histopathological examinations were also performed that clearly depict the restoration of joint cartilage, integrity of leukocytes in synovial fluid and expansion of joint spaces by *I. batatas* extracts.On the other hand, extensive cartilage destruction, narrowing of joint spaces and infiltration of leukocytes in synovial region were observed in arthritic control. The extracts possibly enhanced the expression of extracellular matrix like collagen helix fibers and proteoglycan (PrG), which play extensive role in cartilage restoration.

Reduction of total body weight is an important parameter to find the extent and progression of disease. Altered weight of vital organs like liver, kidney, thymus and spleen is helpful in assessment of various ailments. In the current study, total body weight and indices of the vital organs were measured. The normal and sample treated rats showed a gain of weight, while arthritic animals were found to significantly loose body weight. Likewise, altered organ indices were observed in diseased and test groups in comparison to the normal rats (Fig. 6a, b). Body weight and organ indices based study was also done by Jiang et al. [64] who found positive correlation between body weight variation and arthritis. Atrophy of the liver and thymus in ibuprofen group in the current study clearly suggests its toxicity in multi-dose treatment while the *I. batatas* extracts showed no such deleterious effects. This strengthens our claim of *I. batatas* a better alternative antiarthritic candidate with less or no side effects.

An increased number of the WBCs and ESR in arthritic animals might be due the immune response of pathogenic microorganisms. The decreased RBCs and Hb levels show the anemic condition of arthritic rats (Table 7). ESR is an important hematological tool to index diagnostic or prognostic assessment of inflammatory diseases. Nutritious components like vitamin B, β-carotene, iron, calcium, zinc, and protein are abundantly present in *I. batatas* [23]. The plant is a rich source of anthocyanin and polyphenols responsible for potentiating its ability as antineoplastic, vasotonic, vasoprotective and antiinflammatory agent [25]. The current results are in complete agreement to the abilities reported as they have completely regulated the blood profile of test group in comparison to the arthritic group. *I. batatas* treatment reversed the altered blood parameters in arthritic animals via inhibition of cytokines such as cyclooxygenase and suppression of pro-inflammatory immune response as reported earlier by Hasan et al. [65].

Elevated levels of different serum enzymes, including ALP, ALT, AST and biochemical parameters such as bilirubin indicate abnormality of liver function [66]. These enzymes were monitored to ascertain the normal function of the liver. The significantly elevated serum enzyme shows the toxic response of arthritis in experimental rats (Table 8). High levels of AST, ALT and ALP as well as creatinine and low level of albumin in arthritic rat clearly denote hepatic injuries that were restored in test groups. Hepatic damage is associated with enhanced generation of ROS in hepatocytes that cause cellular death due to DNA damage, protein oxidation and lipid peroxidation. Polyphenole abundance imparts *I. batatas* an ability to scavenge free radicals, which ultimately reduces ROS and hepatocellular death. Batool et al. [42] also mentioned that polyphenols imparts hepatoprotective potential to the plants.

Endogenous antioxidant enzymes, including CAT, POD and SOD protect the oxidation induced cartilage destruction in inflammatory conditions such as rheumatoid arthritis. Inconsistency of the oxidative system in arthritis promotes and intensifies cellular response and increase the destruction of cartilage and bone [33]. When ROS establishes a stress condition, endogenous antioxidants provide protection against the invading free radicals to cope the stressed situation. Administration of an exogenous antioxidant source like *I. batatas* not only scavenges abundant free radicals by oxidizing them but also restore the amount of natural antioxidants. In this study, the enormous production of free radicals with the help of CFA generated an oxidative stress that extensively damaged the liver. This reduced the expression of antioxidative enzymes in arthritic control. On the other hand, polyphenol rich *I. batatas* extracts provided a fine line of defense to the rat liver against ROS. The current study is in agreement to the findings of Khan et al. [67] and Kazmi et al. [68]. Moreover, *I. batatas* extracts provided significant inhibition of free radicals in vitro*,* which fortifies their capabilities to provide a better line of defense in in vivo. *I. batatas* extracts are rich in antioxidant compounds that are the major factors of its ability to scavenge the blend of free radicals continuously produced in disease condition like arthritis and provide a real time protection.

All the phytochemical, in vitro and in vivo results clearly depict the therapeutic worth of *I. batatas* extracts against inflammatory disorder like arthritis. It strongly lessened the inflammation, increased thresholds of pain, enhanced the levels of endogenous enzymes, maintained normal blood profile and upheld the levels of liver and kidney enzymes.

## Conclusion

Multi-mode phytochemical and pharmacological evaluation of *I. batatas* L. Lam. justifies its medicinal worth as a potential agent for curing acute and chronic inflammatory arthritis. High quantity of potent polyphenols along with massive quantity of dietary components makes this food plant a safe and effective candidate in therapeutic rivalry of finding newer potential agents. The histological restoration, body weight and organ weight index stabilization, hematological and biochemical repair as well as endogenous enzymes revitalization clearly defend the benefit of *I. batatas* in arthritis condition. High percentage of inflammation inhibition by the roots of *I. batatas* clearly suggests detailed evaluation of the plant at molecular level. The plant already in use as an antiinflammatory agent in folk medicine is for the first time investigated and presented as an effective antiarthritic agent with good recovery from acute and chronic stages of the disease.

## Additional files

Additional file 1: **Figure S1b.** Chondrocytes exposed to different concentrations of the IPT-EA, IPT-M, IPR-EA and IPR-M for 24 h and cell viability/toxicity was investigated through MTT assay. Results are mean of triplicate experiment ±SD. (JPG 140 kb)

Additional file 2: **Figure S2b.** In vitro antioxidant activities assessment (A) DPPH radical scavenging activity (B) Nitric oxide scavenging activity (C) Hydroxyl radical scavenging activity (D) iron chelating % inhibition. Each value represents mean ± SD (*n* = 3). (JPG 191 kb)

## Abbreviations

ALP: Alkaline phosphatase
ALT: Alanine aminotransferase
AST: Aspartate Aminotransferase
CFA: Complete Freund’s Adjuvant
IPR-EA: *Ipomea batatas* root ethyle acetate extract
IPR-M: *Ipomea batatas* root methanol extract
IPT-EA: *Ipomea batatas* tuber ethyle acetate extract
IPT-M: *Ipomea batatas* tuber methanol extract
MTT: methylthiazole tetrazolium
OECD: Organization for Economic Cooperation and Development
POD: Peroxidase
PrG: Proteoglycan
SOD: Superoxide dismutase
