# Supplementary figures and images for: Ipomoea batatas L. Lam. ameliorates acute and chronic inflammations by suppressing inflammatory mediators, a comprehensive exploration using in vitro and in vivo models
Source: BMC Complement Altern Med. 2018 Jul 13;18:216. doi: 10.1186/s12906-018-2279-5 (PMC6045844; doi:10.1186/s12906-018-2279-5)

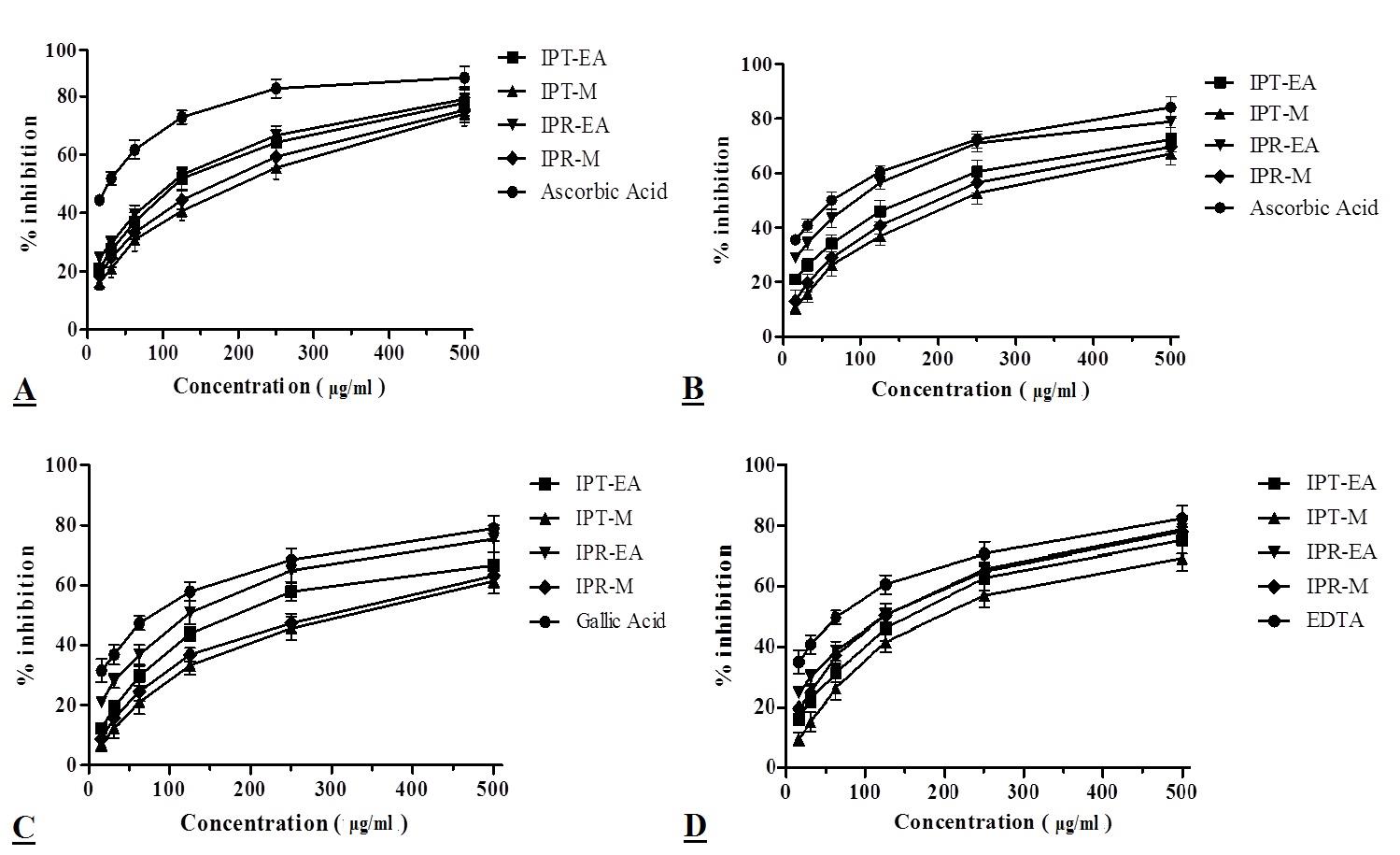

Supplement: Supplementary file 1 — Figure S1b. Chondrocytes exposed to different concentrations of the IPT-EA, IPT-M, IPR-EA and IPR-M for 24 h and cell viability/toxicity was investigated through MTT assay. Results are mean of triplicate experiment ±SD. (JPG 140 kb) [file 12906_2018_2279_MOESM1_ESM.jpg]

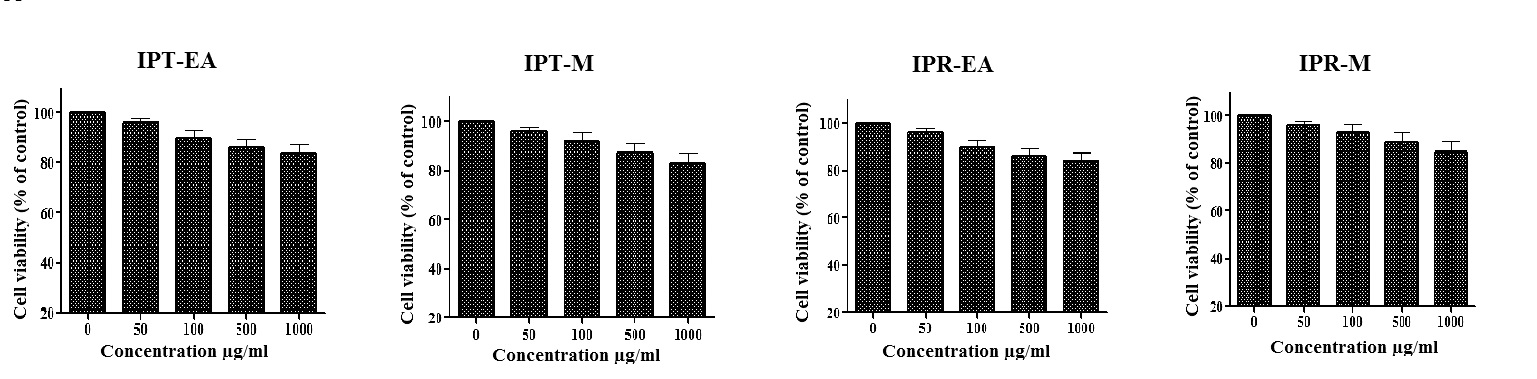

Supplement: Supplementary file 2 — Figure S2b. In vitro antioxidant activities assessment (A) DPPH radical scavenging activity (B) Nitric oxide scavenging activity (C) Hydroxyl radical scavenging activity (D) iron chelating % inhibition. Each value represents mean ± SD (n = 3). (JPG 191 kb) [file 12906_2018_2279_MOESM2_ESM.jpg]
